# CXCR4 Inhibition Enhances the Efficacy of CD19 Monoclonal Antibody-Mediated Extermination of B-Cell Lymphoma

**DOI:** 10.3390/ijms26052024

**Published:** 2025-02-26

**Authors:** Nupur Khunti, Manish Kumar, Moumita Datta, Jean de Dieu Harelimana, Mirja Harms, Dan Albers, Frank Kirchhoff, Jan Münch, Steffen Stenger, Christian Buske, Palash Chandra Maity

**Affiliations:** 1 Institute of Experimental Cancer Research, Ulm University Medical Center, 89081 Ulm, Germanymanish.kumar@uni-ulm.de (M.K.);; 2 Institute of Immunology, Ulm University Medical Center, 89081 Ulm, Germany; moumita.datta@uni-ulm.de; 3 Institute of Microbiology and Hygiene, Ulm University Medical Center, 89081 Ulm, Germany; 4 Institute of Molecular Virology, Ulm University Medical Center, 89081 Ulm, Germany

**Keywords:** B-cell lymphoma, Waldenström Macroglobulinemia (WM), CD19, monoclonal antibody (mAb), CXCR4

## Abstract

CD19 and CXCR4 are pivotal regulators of B-cell activation and migration, respectively. Specifically, CXCR4 signaling critically influences the dissemination of various malignant B cells through constitutive activation and aberrant expression. This study explores the interaction between CD19 and CXCR4 signaling in the context of B-cell lymphomas, particularly focusing on diffuse large B-cell lymphoma (DLBCL) and Waldenström Macroglobulinemia (WM). We assessed the roles of CD19 in survival and CXCL12-induced migration by using knockout (KO) cells of DLBCL and WM origin alongside evaluating the impact of CD19 monoclonal antibodies (mAbs) on antibody-dependent cell-mediated cytotoxicity (ADCC). Our results highlight that CD19 is important for survival and CXCL12-induced migration, and mAbs variably increase CXCL12-induced migration and enhance ADCC. Additionally, we demonstrate that the endogenous peptide inhibitor of the CXCR4 (EPI-X4) derivative JM#21 effectively inhibits CD19-mediated migration enhancement and promotes ADCC, thereby augmenting the therapeutic efficacy of CD19 mAb-based immunotherapy in lymphoma models. Our study underscores the potential of targeting both CD19 and CXCR4 to refine therapeutic strategies for treating B-cell malignancies, suggesting a synergistic approach could improve clinical outcomes in WM treatment.

## 1. Introduction

CD19, an integral member of the immunoglobulin superfamily, serves as a coreceptor together with the B-cell antigen receptor (BCR). As a B-lineage-specific marker, CD19 is expressed throughout the lifecycle of B cells, including in most B-cell lymphomas. While BCR signaling controls B cells’ development and maturation, CD19 generates co-stimulatory activation signals that prevent antibody deficiencies or hypogammaglobulinemia [1,2]. As a critical component of the BCR signal amplifier, CD19 uniquely lacks a natural ligand. However, its cytoplasmic tail contains multiple phosphorylation sites that are crucial for docking various adapter proteins and kinases, such as Lyn, ERK, and PI3K, upon BCR stimulation. These structural features enable CD19 to act as a hub for signal integration and the amplification of downstream cascades essential for B-cell activation and function [3,4]. Upon antigen engagement of BCR, CD19 coordinates the PI3K signaling pathway, which in turn regulates the cellular metabolism, redox balance, and survival fitness of the activated B cells [5]. Consequently, nearly 98% of malignant B cells retain CD19 expression on their surface, underscoring its value as a promising target for immunotherapy. Currently, CD19-targeted immunotherapy is an alternative to the classical chemoimmunotherapy regimen that combines the cytolytic anti-CD20 antibody rituximab with cyclophosphamide, doxorubicin, vincristine, and prednisone (R-CHOP) [6,7].

CD19-targeted immunotherapy has significantly improved the treatment options for various B-cell malignancies, spanning aggressive Diffuse Large B-cell Lymphoma (DLBCL) to the more indolent Waldenström Macroglobulinemia (WM). Particularly, those cases that are non-responsive, refractory, or have relapsed (R/R) from the R-CHOP regimen, accounting for approximately 40% of cases, benefit from anti-CD19-based therapeutics [8]. Therapeutic approaches range from humanized Fc-modified monoclonal antibodies (mAbs) to antibody–drug conjugates and chimeric antigen receptor T cells (CAR-Ts) [9]. Engineered anti-CD19 monoclonal antibodies (CD19 mAbs) with enhanced cytolytic activity are commonly used as B cell-depleting therapies for treating R/R cases of different B-cell lymphomas as well as autoimmune diseases [10,11]. These functionalized mAbs recruit phagocytes like natural killer (NK) cells or Macrophages (Mφ). As a result, the treatment efficacy of CD19 mAbs largely depends on the number of recirculating immune cells and tumor-invading effector cells and their activation states. Thus, a lack of infiltrating effector cells impairs the targeting of tissue-resident malignant B cells, in many instances leading to the unexpected development of escape mechanisms such as the loss of CD19 from the cell surface [12,13].

Most B-cell non-Hodgkin lymphomas, including DLBCL, are characterized by dissemination already at diagnosis. Dissemination of malignant B cells involves chemokine receptor responsiveness, cytoskeletal remodeling, and migration, and all those are critically regulated by BCR and CD19 signaling [14]. Among the chemokine receptors, the C-X-C chemokine receptor type 4 (CXCR4) is notably prominent in many B-cell lymphomas [15]. CXCR4 is a key receptor that binds to the chemokine CXCL12 (also known as stromal cell-derived factor 1, SDF-1), playing a crucial role in the homing and retention of hematopoietic stem cells within the bone marrow. This signaling pathway has gained particular importance due to its aberrant upregulation upon treatment with common BCR signaling inhibitors that target PI3K, BTK, and SYK kinases [16,17,18]. The upregulation of CXCR4 contributes to the increased migratory and invasive capabilities of lymphoma cells, facilitating their spread and complicating treatment. Additionally, dysregulated CXCR4 expression and a C-terminally truncated constitutive active CXCR4 mutant are major prognostic biomarkers for the relatively indolent lymphomas such as the germinal center B cell type (GCB)-DLBCL and lymphoplasmacytic lymphoma (LPL) or WM [15,19,20], respectively.

We have shown that CXCR4 signaling is activated by a BCR cascade and a CD19 signaling module in mouse models [21,22]. Thus, activation of the CD19 module by the CD19 mAbs could cause enhanced BCR and CXCR4 signaling, impacting growth and migration in malignant B cells. However, CD19–CXCR4 signaling axes remain unexplored in human B cells, especially in various B-cell malignancies. The current advancements in anti-CD19-based immunotherapy, including CAR-T, revolutionized the treatment of different B-cell lymphomas. Unfortunately, around 50% of anti-CD19 immunotherapies, including CAR-T treatment, ultimately fail [23]. The proposed theories, such as T-cell exhaustion, the immunosuppressive tumor microenvironment, and escape through antigen suppression, could not fully explain the relapse mechanism. In this context, CD19–CXCR4 signaling effect could be more aggravated when there is an inadequate number of recirculating effector cells or when the CD19 mAbs fail to recruit cytolytic natural killer (NK) cells and induce antibody-dependent cell-mediated cytotoxicity (ADCC). Consistent with our hypothesis, a recently developed nondepleting CD19 mAb, LY3541860, which readily inhibits B-cell activation, proliferation, and differentiation independent of NK cell recruitment, demonstrates improved efficacy over B-cell depletion therapy in autoimmune disease models [24]. Presumably, the nondepleting and inhibitory CD19 mAb impairs CD19–CXCR4 signaling crosstalk even with the lack of tumor-invading effector cells and prevents the further dissemination of malignant B cells. However, the role of CD19 in promoting CXCL12-induced migration and survival of lymphoma cells remained largely undetermined.

To investigate this, we studied the role of CD19 and the effect of CD19 mAbs on CXCR4 signaling in GCB-DLBCL and WM models using the SU-DHL-6 (hereafter called DHL6) and BCWM.1 cell lines, respectively. Moreover, we tested the differences in anti-CD19 mAb clonotypes causing CXCR4 stimulation and their cytolytic abilities by testing the effect on migration and efficacy in ADCC, respectively. We showed that CD19 signaling is required for the CXCL12-induced migration and survival of lymphoma cells. Next, we utilized CXCR4 peptide antagonists to impair CD19–CXCR4 crosstalk. Altogether, our data show that a selective CXCR4 peptide antagonist improves on CD19 mAb for targeted immunotherapy against DLBCL and WM.

## 2. Results

### 2.1. Generation and Characterization of CD19 Knockout Lymphoma Cell Lines

To test our hypothesis that CD19 is important for CXCR4 signaling and the overall survival of WM cells, we generated CD19 knockout (KO) BCWM.1 cells using CRISPR-Cas9 methods (Figure S1). In parallel, we also generated IgM KO BCWM.1 cells to investigate the influence of BCR on the survival of these cells. We adopted a two-step strategy that supports CRISPR-Cas9 engineering both through lentivirus (Biosafety Level 2; BSL2) and murine ecotropic γ-retrovirus (BSL1)-based delivery of the targeting sgRNA (see Section 4). Supporting our hypothesis, cells transduced with the CD19-targeting sgRNA accompanying a GFP reporter were outcompeted by the untransduced cells and partly lost from the mixture between 7 and 15 days post transfection and prior to single-cell sorting (Figure S1A). Therefore, we first sorted all GFP-positive transduced cells in bulk, grew them for few days, and then sorted them as single cells for generating clones. The average numbers of growing clones were significantly lower for the CD19 KO cells compared with the wildtype (WT) cells (Figure S1B). In the case of IgM KO, the effect was even stronger. Upon collecting the growing CD19 KO BCWM.1 clones, we first confirmed the loss of CD19 expression in these cells by flow cytometry (Figure S1C) and determined the mutation in the CD19 locus by sequencing (Figure S1D). Compared with the WT BCWM.1 cells, the CD19 KO cells did not show any differences in the surface expression of IgM-BCR and CXCR4 (Figure S1E) or in IgM secretion (Figure S1F), suggesting no autoregulation and compromised receptor expressions in this model. Following the same method, we also generated CD19 KO of GCB-DLBCL-derived DHL6 cells and characterized them (Figure S1G,H).

### 2.2. CD19 Is Required for Growth and CXCL12 Induced Migration of Lymphoma Cells

**Figure 1:**
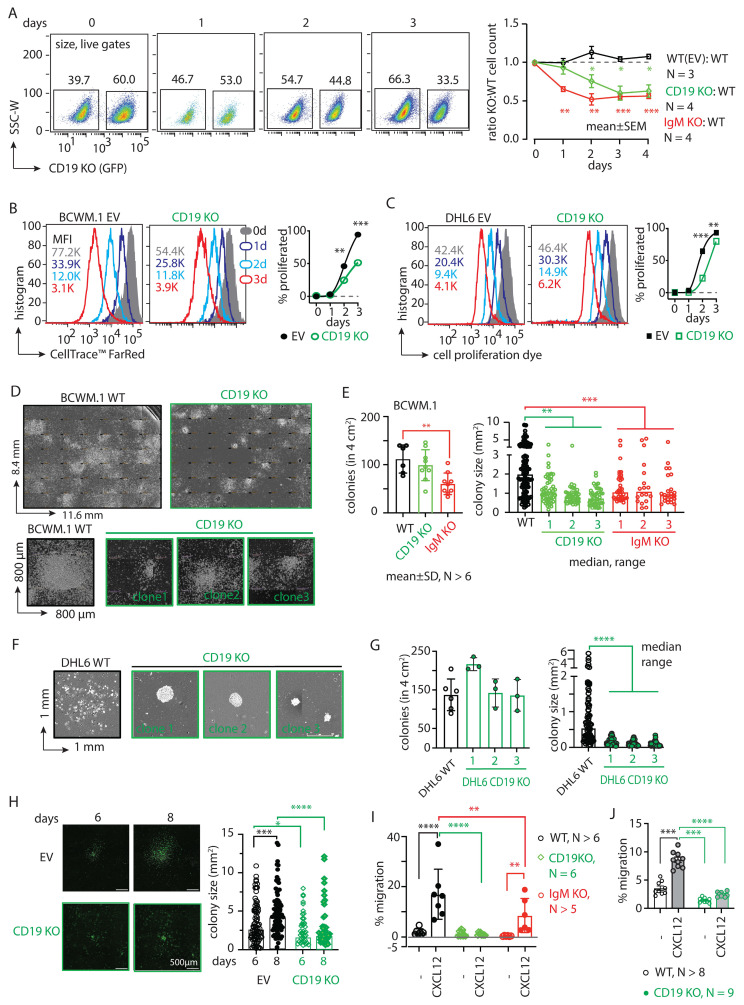
CD19 is essential for the survival and migration of lymphoma cells. (**A**) Left, FACS analyses showing percentage changes in living populations of CD19 KO (GFP positive) BCWM.1 cells compared with WT cells at 1–3 days after seeding the coculture. Cells were gated for size by FSC-A and SSC-A followed by live–dead Sytox™ Blue staining. Right, time kinetics showing changes in the ratio of CD19 KO:WT (green) cell numbers compared with EV:WT (black) or IgM KO:WT (red), obtained from the FACS analyses of the coculture (as depicted in Figure S1J). (**B**) Left to right, representative histogram overlay of cell proliferation assay after 0, 1, 2, and 3 days after staining BCWM.1 WT (**left**) and CD19 KO (**middle**) with 0.5 µM of CellTrace™ FarRed, followed by time kinetics showing changes in the percent of proliferated cells (**right**). Mean fluorescence intensity (MFI) values are indicated within the plots. (**C**) Same as (**B**), using DHL6 WT and CD19 KO cells. (**D**) Ensembled microscopic images of 10-days CFA assay showing an overview of colonies formed by WT BCWM.1 cells compared with the representative CD19 KO clone (green). For comparison of individual colony features, single colonies from three independent representative clones of CD19 KO BCWM.1 cells are shown below. Image was produced in Fiji (ImageJ2 version 2.14.0/1.54f) by stitching a grid of overlapping brightfield images taken at 10× magnification. Dimension of the images are indicated in their axes. (**E**) Quantification of the number of colonies (**left**) obtained in a 4 cm^2^ well and the estimated colony (area) size (**right**) of the individual colonies obtained from a minimum of three independent clones for CD19 KO and IgM KO compared with WT BCWM.1 cells. (**F**) Representative images of single colonies from three independent representative clones of CD19 KO DHL6 cells compared with WT cells. (**G**) Quantification of the number of colonies (**left**) and colony size (**right**) of a minimum of three independent CD19 KO clones compared with WT DHL6 cells. (**H**) Live cell fluorescence images of EV (GFP)-transduced WT and CD19 KO (GFP positive) BCWM.1 cells representing a single growing colony at days 6 and 8 and the corresponding quantifications of colony sizes. (**I**) Specific migration of CD19 KO and IgM KO clones toward CXCL12 (60 nM) compared with WT BCWM.1 cells. (**J**) Same as (**I**), specific migration of CD19 KO DHL6 clones compared with WT control. Data in (**A**–**C**,**I**,**J**), and colony counts in (**E**,**G**) represent mean ± SD of indicated N number of replicates and were analyzed by Two-Way ANOVA followed by Dunnett’s multiple comparison for each pair with reference to control. Colony size data (**E**,**G**,**H**) are median ± range and were analyzed by One-Way ANOVA followed by Dunn’s multiple comparison or directly by the Mann–Whitney test, respectively. * *p* < 0.05, ** *p* < 0.01, *** *p* < 0.001, **** *p* < 0.0001.

To investigate the survival competence of CD19 KO BCWM.1 cells in competition with the WT cells, we cocultured them at a 1:1 ratio and measured cell growth by counting the cell numbers with flow cytometry (Figure S1I). Expectedly, the CD19 KO clones were slow growing and were ~40% lost (surviving fractions on days 2–4: 75.5 ± 16.9; 60.1 ± 18.3; and 62.8 ± 15.9) in competition with their WT counterparts within 3–4 days of coculturing (Figure 1A). The IgM KO cells, on the other hand, more rapidly declined to <60% (days 2–4: 52.1 ± 14.9; 55.5 ± 6.7; 56.3 ± 6.5) within 2 days (Figure 1A), indicative of the stronger BCR signaling dependence of lymphoma cells. In addition, we directly assessed the proliferation up to three days using a dye dilution assay (Figure 1B,C), which illustrated a decrease in the cell division rate for both BCWM.1 and DHL6-derived CD19 KO cells compared with their WT counterparts. Overall, these data suggest that both CD19 and IgM play crucial roles in the survival and overall growth of BCWM.1 cells.

Next, we assessed the colony-forming ability (CFA) of these cells by culturing them in a methylcellulose-based matrix and monitored the colony formation of the BCWM.1 and DHL6 cells (Figure 1D–H). Compared with WT cells, the CD19 KO clones grew significantly slower and produced smaller colonies (Figure 1D–G). While the number of colonies produced by the BCWM.1 CD19 KO clones (mean ± SD, 100 ± 32) was unchanged compared with WT (103 ± 29), significantly reduced numbers of colonies were produced by the IgM KO (59 ± 22) BCWM.1 clones (Figure 1E). Like WM, the loss of CD19 in the GCB-DLBCL derived DHL6 cells also caused reduced colony size, without changes in the number of colonies (Figure 1F,G). Notably, we failed to generate IgM KO DHL6 cells, suggesting an indispensable role of BCR signaling for the survival of DLBCL cells [12,25]. Surprisingly, the total counts of CD19 KO BCWM.1 and DHL6 cells after ten days of CFA were only marginally reduced or remained unchanged compared with those of the WT controls (Figure S2A). This suggests a specific role of CD19 in cell proliferation and colony growth, whereas IgM simply prevents colony formation due to severe survival disadvantage and produces significantly reduced numbers of colonies. To understand this difference in proliferation rates, we analyzed Ki-67 expression, a widely known marker of cell proliferation used to determine clinical aggressive behavior in related lymphomas such as mantle cell lymphoma [26,27]. In both the BCWM.1 and DHL6 derived CD19 KOs, Ki-67 expressions were significantly reduced compared with WT counterparts (Figure S2B,C). Surprisingly, upon CD40 ligand (CD40L) treatment that induces the proliferation of all B cells, the CD19 KOs did not increase Ki-67 expression. Intriguingly, a colony replating assay confirmed the smaller, condensed colonies of CD19 KO cells compared with WT BCWM.1 cells (Figure S2D), suggesting a role of CD19 in intra-colony cell mobilization, spreading, and subsequent growth of colony size.

We therefore performed live cell imaging to monitor the colony formation and spreading of BCWM.1 cells over time (Figure 1H). Like the CFA assay, we plated CD19 KO BCWM.1 cells and empty vector (EV)-transduced GFP-positive control cells in a methylcellulose-based matrix and monitored the cell growth every 24 h by real-time imaging (see Section 4). Between 6 and 8 days, the colonies became visible and spread around the proliferation center. As depicted in Figure 1H, CD19 KO BCWM.1 cells failed to grow and spread, resulting in smaller, condensed colonies compared with the EV-transduced cells.

Next, we analyzed the CXCR4 response in CD19 KO cells by testing CXCL12-induced migration. Both BCWM.1 and DHL6 failed to migrate in response to CXCL12 upon the loss of CD19 (Figure 1I,J and Figure S2E). IgM KO BCWM.1 cells, on the other hand, exhibited only partial reduction in CXCL12-induced migration, suggesting differential roles of CD19 and BCR in CXCR4 signaling (Figure 1I). To establish the optimal migration rates for both BCWM.1 and DHL6, various dosages of CXCL12 (ranging from 15 to 240 nM) were analyzed, identifying the most effective conditions at 60 and 120 nM of CXCL12, respectively (Figure S2E). Notably, the rate of specific migration by WT DHL6 cells (no cytokine control, 3.3 ± 1.5%; 60 nM CXCL12, 9.7 ± 1.5%) was much lower than BCWM.1 cells (no cytokine control, 2.4 ± 2.0%; 60 nM CXCL12, 19 ± 8%) and was below 10%, which could not be improved by increasing the dose of CXCL12 in the migration assay (Figure 1J and Figure S2E). This is in line with the general CXCR4 non-responsiveness of the DLBCL cells [28]. Together, these data suggest a specific role of CD19 in cell growth, colony spreading, and CXCL12-induced migration.

### 2.3. CD19 mAbs Increase Survival and CXCL12-Induced Migration of WM Cells

Knowing the essential role of CD19 in the survival and CXCL12-induced migration of both BCWM.1 and DHL6 lymphoma cells, we tested whether treatment with anti-CD19 monoclonal antibodies (mAbs) had any effect on these cells. To this end, we generated a humanized anti-CD19 monoclonal antibody (mAbo) by cloning the variable sequences from an anti-CD19 hybridoma, and we compared its effects with those of commercially available humanized anti-CD19 mAbs (mAb1 and mAb2) used in therapeutic and preclinical studies (see Section 4). Notably, most therapeutic and preclinical mAbs from commercial sources are available in only limited quantities for research use, with minimal information and no options for experimental modification. Therefore, we sought to produce mAbo in HEK293T cells and purified it using HiTrap Protein G affinity chromatography (Figure S3A). Upon determining the amount and purity by SDS-PAGE and ELISA (Figure S3B,C), we tested the binding specificity of mAbo on human peripheral B cells and BCWM.1 cells compared with non-B cells and CD19 KO BCWM.1 cells, respectively (Figure S3D). In both cases, mAbo specifically detected CD19-positive B cells.

**Figure 2:**
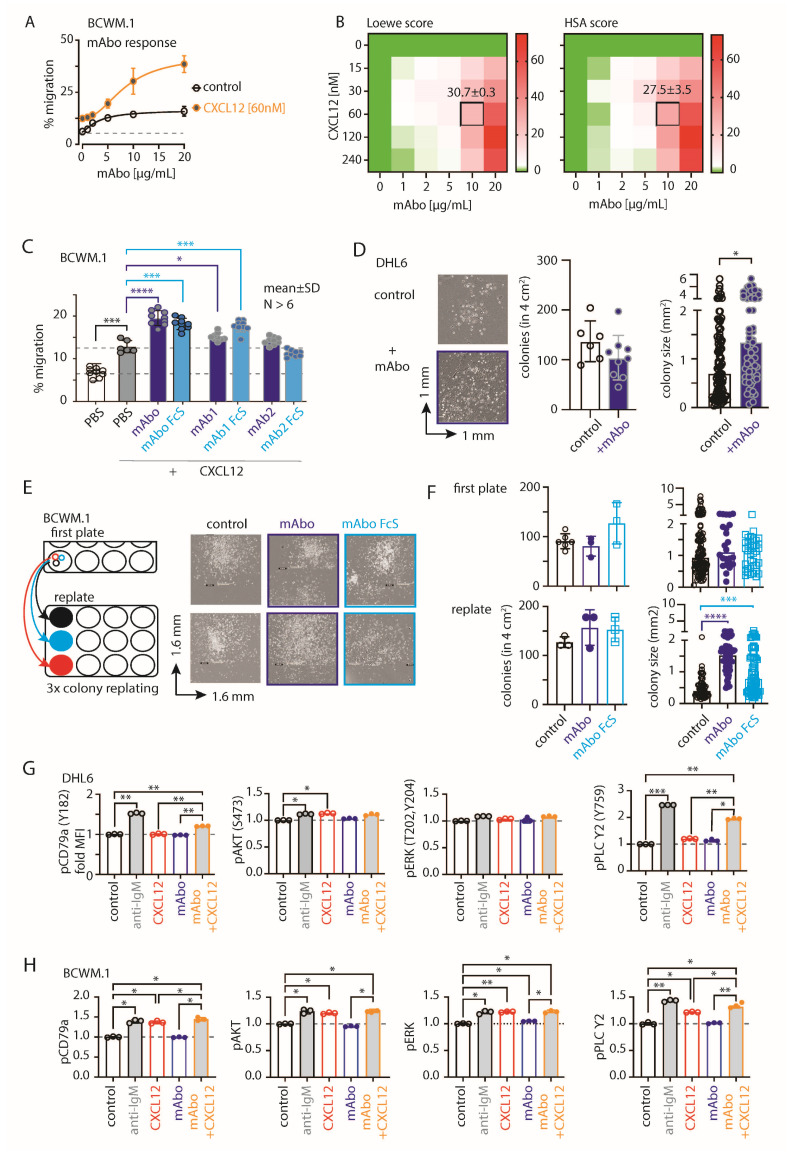
CD19 mAbs increase CXCL12-induced migration and growth. (**A**) Specific migration of BCWM.1 WT cells toward 60 nM CXCL12 compared with no CXCL12 control at varying concentrations of 0, 1, 2, 5, 10, and 20 µg/mL mAbo. (**B**) Heatmaps showing the Loewe (**left**) and HSA (**right**) synergy scores of the combinatorial dose-dependent effects of mAbo1 and CXCL12 on the migration of BCWM.1 WT cells. Plots represent the mean of three experiments analyzed with the SynergyFinder Plus tool. Synergy scores of the selected dose combination, i.e., 10 µg/mL mAbo and 60 nM CXCL12, are indicated inside the plot. (**C**) Specific migration of BCWM.1 cells toward CXCL12 alone (filled gray bar) and in the presence of wildtype anti-CD19 clone mAbo (dark blue bar) and its Fc silent (FcS) counterpart (light blue bar) in comparison with other commercially available therapeutic anti-CD19 clones mAb1 and mAb2 and their FcS forms. Dashed lines represent mean of basal (with PBS) and CXCL12-induced migrations. (**D**) Left panels, representative images of single colonies of CD19 KO DHL6 cells at 10 days of CFC assay in the absence (control) and presence of mAbo. Middle and right panels, quantification of the number of colonies and estimated colony size in the absence and presence of mAbo, respectively. (**E**) Representative images of single colonies of CD19 KO DHL6 cells at 10 days of CFC assay (**upper panels**) and after 8 days of colony replating (**bottom panels**) in the presence of 5 µg/mL anti-CD19 clone mAbo and its FcS form (**F**). Quantification of the number of colonies (**left**) and estimated colony size (**right**) of the individual colonies of CD19 KO BCWM.1 cells in the presence and absence of mAbo or mAboFcS from the first CFC plating and after replating. (**G**) Quantification of pCD79a (Y182), pERK (T202, Y204), pAKT (S473), and pPLCγ2 median fluorescence intensity (MFI) of DHL6 cells (as depicted in Figure S2E) in response to stimulants—5 µg/mL anti-IgM, 60 nM CXCL12, 5 µg/mL mAbo anti-CD19 and mAbo + CXCL12 for 5 min. (**H**) Same as (**G**), quantification of MFIs of stimulated BCWM.1 cells. Data in (**A**–**C**,**G**,**H**) and colony counts in (**D**,**F**) represent mean ± SD indicated number of replicates and were analyzed by One-Way ANOVA followed by multiple comparison with reference to control, except the heatmaps. Colony size data in (**D**,**F**) represent median with range and were analyzed by the Mann–Whitney test. * *p* < 0.05, ** *p* < 0.01, *** *p* < 0.001, **** *p* < 0.0001.

Next, we performed migration assays on BCWM.1 and DHL6 cells with varying dosages from 1 to 20 µg/mL of mAbo (Figure 2A and Figure S3E). The dose-dependent increment in CXCL12-induced migrations suggests a role for activated CD19 signaling crosstalk with CXCR4 signaling. In order to determine the optimum commination, we analyzed the combinatorial dose-dependent migration response of BCWM.1 cells using variable dosages of mAbo and CXCL12, calculated the synergy matrix using the highest single agent (HSA) model, and linearized the additive effect of individual compounds by Loewe’s model (Figure 2B) [29]. As depicted in the Figure 2B heatmaps, both models demonstrate synergic interactions between mAbo and CXCL12 at higher doses of mAbo. Notably, scores > 10 and < −10 represent synergistic and antagonistic interactions, respectively [29,30]. In addition, we tested the same dosages of mAbo on CD19 KO cells, showing no enhancement in CXCL12-induced migration compared with the WT counterpart (Figure S3F). Unlike BCWM.1 cells, mAbo treatment led to less effective migration enhancement in DHL6 cells (Figure 2A and Figure S3E), corroborating with the restricted CXCR4 responsiveness observed in DLBCL cells [28]. Therefore, we selected the dosage combination of 10 µg/mL mAbo and 60 nM CXCL12, which corresponds to Loewe and HSA synergy scores in BCWM.1 cells at 30.7 ± 0.3 and 27.5 ± 3.5, respectively (Figure 2B). To compare the enhanced migration by mAbo, we used two other commercial humanized CD19 clones, mAb1 and mAb2 (see Section 4). The mAb1 clone effectively increased CXCL12-induced migration similar to our mAbo. In contrast, the mAb2 clone minimally affected the migration, suggesting differential activation responses through CD19 antibody clones (Figure 2C). Additionally, we tested the Fc silent (FcS) mutant versions of all these humanized mAbs to avoid aberrant Fc receptor (FcR)-mediated recruitment. In all cases, the FcS versions behaved similarly to the unmutated mAbs, thereby eliminating interference through FcR on the B cell surface. When tested on CD19 KO BCWM.1 cells, none of these mAbs altered CXCL12-induced migration (Figure S3G). In parallel to migration, we assessed the effect of mAbo on colony formation (Figure 2D,E). While DHL6 colony sizes significantly increased upon the addition of mAbo (Figure 2D), BCWM.1 colonies exhibited a trend toward increase only after 10 days (Figure 2E). Therefore, we replated the colonies to allow them to grow on a new surface, where the BCWM.1 colonies were found to significantly increase in size in response to mAbo treatment (Figure 2E). Notably, mAbo treatment did not affect the number of colonies, suggesting no impact on colony seeding or initiation. Instead, it led to enhanced proliferation and spreading, resulting in larger colonies.

Next, we analyzed the CXCR4 and BCR proximal phosphorylation signaling using an intracellular phospho-flow cytometry assay (Figure 2G,H and Figure Figure S3H). To optimize the assay conditions, we tested phosphorylation signals for pCD79a (Y182), pERK (T202, Y204), pAKT (S473), and pPLC-γ2 (Y759) in DHL6 cells following anti-IgM or CXCL12 treatment for 5 and 10 min (Figure S2H). While BCR stimulation with anti-IgM readily increased the phosphorylation of all tested markers, CXCL12 treatment selectively induced pAKT and pPLC-γ2 in DHL6 cells (Figure 2G and Figure S3H). Notably, DHL6 cells were only minimally responsive to CXCL12-induced migration as compared with BCWM.1 (Figure 1J and Figure S2E). Interestingly, the combination of mAbo and CXCL12 treatments resulted in a greater increase in pPLC-γ2 and pCD79a, suggesting a synergistic CXCR4 signal amplification through CD19. Similar results were observed for BCWM.1 cells (Figure 2H). In contrast to DHL6 cells, CXCL12 treatment alone induced phosphorylation of all tested markers in BCWM.1 cells [31], reaching levels comparable to those induced by anti-IgM treatment. This suggests differential effects of BCR and CXCR4 signaling between WM and DLBCL.

### 2.4. Variable Efficiencies of CD19 mAbs in Inducing ADCC

**Figure 3:**
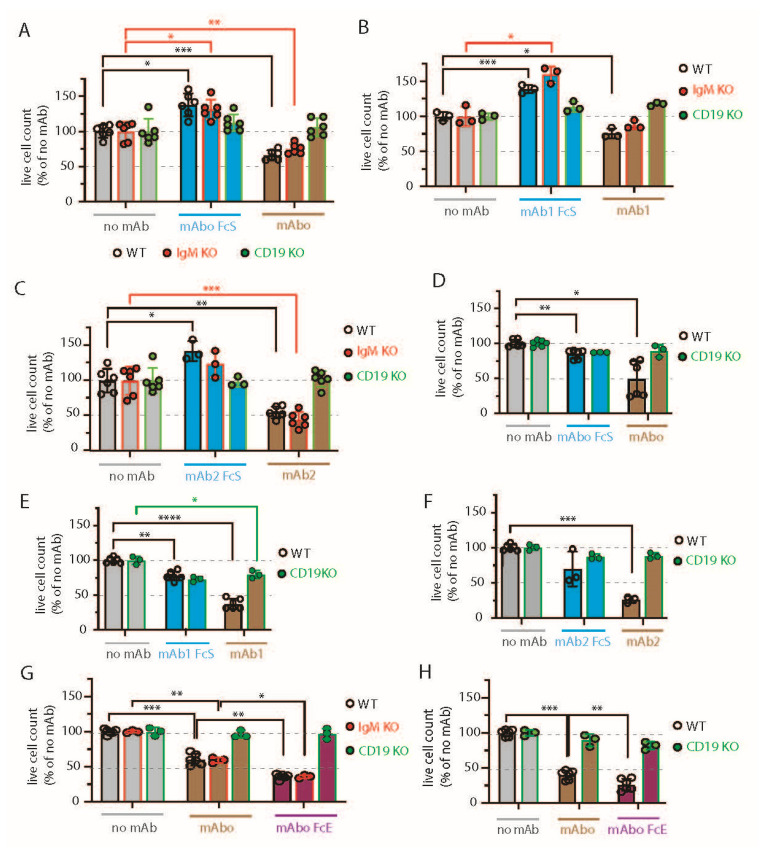
Variable efficiencies of CD19 mAb-induced ADCC on different lymphoma cells. (**A**) Quantification of WT (black, open circles), IgM KO (red, solid circles), and CD19 KO (green, solid circles) BCWM.1 live cell count in ADCC assay (as depicted in Figure S3) in the absence (gray bars) and presence of mAboFcS mutant (blue bars) or mAbo (brown bars) as indicated. Plots represent mean ± SD of three or more replicate data normalized to no mAb control and were analyzed by Two-Way ANOVA followed by Dunnett’s multiple comparison. * *p* < 0.05, ** *p* < 0.01, *** *p* < 0.001, **** *p* < 0.0001. Dashed lines represent 100 and 50% survival values. (**B**,**C**) Same as A, ADCC assay with mAb1FcS vs. mAb1 (**B**) and mAb2FcS vs. mAb2 (**C**). (**D**–**F**) Same as (**A**), live cell counts of WT (black, open circles) and CD19 KO (green, solid circles) DHL6 cells upon ADCC assay with mAboFcs vs. mAbo (**D**), mAb1Fcs vs. mAb1 (**E**), and mAb2Fcs vs. mAb2 (**F**). (**G**) Effect of Fc binding enhanced (FcE) anti-CD19 clone mAboFcE (purple bars) on WT (black, open circles), IgM KO (red, solid circles), and CD19 KO (green, solid circles) BCWM.1 cells in ADCC assay compared with no mAb (gray bars) and standard mAbo (brown bars) controls. (**H**) Same as (**G**), enhanced ADCC of mAboFcE on DHL6 cells compared with standard mAbo control.

The clinical success of anti-CD19 mAbs relies on B cell-depleting cytolytic activity, specifically through antibody-dependent cell-mediated cytotoxicity (ADCC) in the presence of activated natural killer (NK) cells [32]. We therefore evaluated the efficacy of the CD19 mAbs in ADCC in the presence of IL-2 stimulated NK cells isolated from human peripheral blood (Figure 3 and Figure S4). We optimized an FACS-based quantitative analysis to determine the absolute live cell count and to distinguish cell types based on endogenous markers post ADCC assay (Figure S4A–E). Briefly, cell numbers were normalized to the number of live lymphoma cells obtained from the 4 h control experimental ADCC coculture of lymphoma and rhIL-2 activated NK cells without any mAb addition (no mAb, Figure S4C). Expectedly, we found that ADCC in the presence of mAbo reduced the WT BCWM.1 cells to 68.9 ± 7.2% in comparison with no mAb control, 100.4 ± 9.4% (Figure 3A and Figure S4C–E). Similarly, live IgM KO BCWM cells were reduced from 108.7 ± 14.6% in the no mAb control to 81.6 ± 7.8% due to mAbo-induced ADCC. In contrast, CD19 KO cells remained unaffected by mAbo-induced ADCC in the presence of activated NK cells. Similarly, the mAboFcS treatment failed to kill any of the WT and KO BCWM.1 cells due to a lack of NK cell engagement through FcR (Figure 3A and Figure S4C,D). Interestingly, normalized percentages of living WT and IgM KO BCWM.1 cells treated with mAboFcS were increased to 143.6 ± 17% and 142.8 ± 15.4%, respectively. This result supports our hypothesis that the failure to ligate a cytotoxic NK cell could potentially increase the survival of lymphoma cells in the presence of CD19 mAbs. Notably, the absolute lymphoma cell counts decrease for all BCWM.1 and DHL6 cell types in the presence of activated NK cells in 4 h control experimental ADCC coculture without any mAb (Figure S4B,C,F). This systemic loss of cell survival under control experimental ADCC coculture is prevented by the addition mAboFcS treatments (Figure 3A and Figure S4F).

Similar ADCC responses against different BCWM.1 cell types were seen for the other two clonotypes of CD19 antibodies mAb1 and mAb2 and their corresponding FcS forms, mAb1FcS and mAb2FcS (Figure 3B,C). While the mAb1 clone reduced the WT BCWM.1 cells to 75.8 ± 7% only, mAb2 treatment reduced them to 54.8 ± 8.3% survival, suggesting a variable response to different mAb clonotypes. Similarly, mAb1- and mAb2-induced ADCC reduced the IgM KO cells to 87.5 ± 6.2% and 44.3 ± 11.4% (Figure 3B,C), respectively. As control, there was no effect of these clonotypes on CD19 KO cells. Interestingly, both mAb1FcS and mAb2FcS caused a significant increase in the survival of WT BCWM.1 cells up to 138.9 ± 5.8% and 142.5 ± 14.2% (Figure 3B,C), respectively. Unlike WT and IgM KO cells, there was no increase in survival of CD19 KO BCWM.1 cells upon mAb1FcS or mAb2FcS treatment, demonstrating the role of activated CD19 signaling in survival advantage.

For DHL6 cells, all three different CD19 mAb clones showed relatively higher ADCC efficacy compared with BCWM.1 cells (Figure 3D–F). The percentages of WT DHL6 that survived in ADCC upon mAbo, mAb1, and mAb2 treatments were 49 ± 25.4, 37.4 ± 7.4, and 25.8 ± 4.5, respectively. In contrast to the effects on BCWM.1 cell types, all three clonotypes in their FcS forms failed to cause any survival advantage for DHL6 cells compared with no mAb treatment (Figure 3D–F and Figure S4F). In other words, reverting the systemic loss of cell survival under control experimental ADCC coculture was ineffective for DHL6 cells.

To improve on the efficacy of our mAbo clonotype in ADCC, we aimed at generating a mutant with enhanced FcR binding, as described before [33,34]. To this end, we introduced two point-mutations at the IgG1 CH2 domain, namely S239D and I332E, resulting in an mAbo-Fc enhanced (mAboFcE) version. We repeated the ADCC assay on BCWM.1 and DHL6 cells with this mAboFcE and compared it with the original, unmutated mAbo treatment (Figure 3G,H). Expectedly, the mAboFcE treatment enhanced ADCC and drastically reduced survival of WT BCWM.1 cells to 36 ± 3.8% compared with 60.4 ± 8.2% survival upon non-modified mAbo treatment (Figure 3G). Similarly, survival of WT DHL6 cells was reduced to 26.3 ± 8.4% by mAboFcE treatment compared with 40.2 ± 6.7% upon non-modified mAbo treatment (Figure 3H). As a control, there was no effect of mAboFcE on any CD19 KO cell types. Altogether, these data show differential effects of CD19 mAb clonotypes on ADCC response against lymphoma cells, with the highest efficacy caused by the mAb2 clonotype against both WM and DLBCL. The clonotype-specific variations were more pronounced for BCWM.1 compared with DHL6 cells, which is indicative of their relative CXCR4 signaling dependency (Figure 2C). Furthermore, by creating the enhanced FcR binding mutant mAboFcE, we could improve on our in-house-generated anti-CD19 antibody and attain the similar efficacy as the mAb2 clonotype (Figure 3C,G).

### 2.5. CXCR4-Antagonizing Peptide Enhances CD19 mAbs-Induced Enhanced Migration

**Figure 4:**
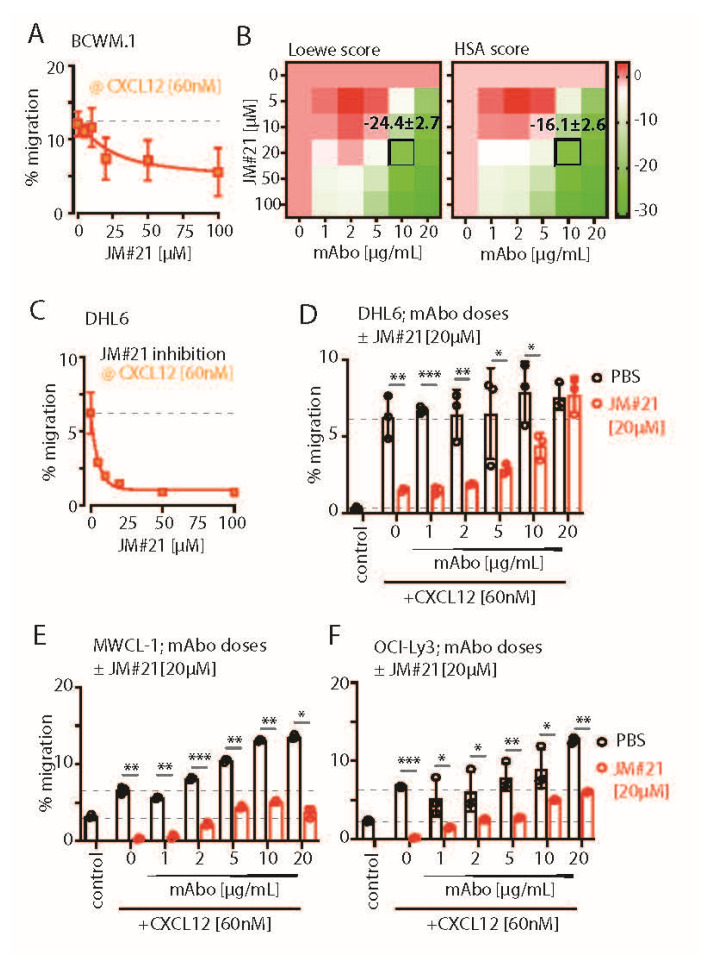
CXCR4-ntagonizing peptide JM#21 inhibits CD19 mAbo-induced enhanced migration. (**A**) Dose-dependent decrease in the specific migration of BCWM.1 WT cells toward 60 nM CXCL12 at varying concentrations of 15, 30, 60, 120, and 240 nM CXCR4 antagonist JM#21. Dashed line represents % migration mean in absence of JM#21. (**B**) Heatmaps showing the Loewe (**left**) and HSA (**right**) synergy scores of combinatorial dose-dependent effects of mAbo1 and JM#21 on the migration of BCWM.1 WT cells. Plots represent the mean of three experiments analyzed with the SynergyFinder Plus tool. Synergy scores of the selected dose combinations, i.e., 10 µg/mL mAbo and 60 nM CXCL12, are indicated inside the plot. (**C**) Same as (**A**), specific migration of DHL-6 cells at varying concentrations of JM#21. (**D**–**F**) Specific migration of DHL-6, MWCL.1, and OCI-Ly3 cells toward 60 nM CXCL12 at varying concentrations of mAbo in the absence and presence of 20 µM JM#21. Dashed lines represent mean of basal (with PBS) and CXCL12-induced migrations in absence of mAbo. Data represent mean ± SD of three experiments and were analyzed by Two-Way ANOVA followed by Šídák’s multiple comparisons. * *p* < 0.05, ** *p* < 0.01, *** *p* < 0.001.

To down-modulate the CD19 mAb-induced activation of CXCR4 response, we then explored the combinatorial potential of CXCR4 inhibition to improve the mAb-induced ADCC. The endogenous peptide inhibitor of CXCR4 (EPI-X4) specifically antagonizes CXCR4 and is therefore a promising candidate for drug development for the treatment of CXCR4-dependent diseases [35]. Optimized EPI-X4 derivatives reduced the tumor burden in different cancer models, specifically, the survival of the WM cell line in immunocompromised mouse recipients [31,36,37]. We here tested the original EPI-X4 and an optimized version named EPI-X4 JM#21 (herein referred to as JM#21), which showed 1000-fold increased antagonistic activity compared with the WT peptide [37]. In addition, we included the small-molecule CXCR4 antagonist AMD3100 (Plerixafor), which is clinically approved for hematopoietic cell mobilization for autologous stem cell transplantation [38]. Previously, both EPI-X4 and JM#21 treatments were shown to inhibit basal survival signaling pathways by reducing ERK and AKT phosphorylation, with a subsequent loss of survival and apoptosis in WM cells [31]. Therefore, we first tested the dose response of EPI-X4 and JM#21 for 4 h of treatment, as described for the ADCC assay, and measured the cell survival after 12–18 h post removal of inhibitors (Figure S5A). The calculated IC_50_ for EPI-X4 and JM#21 treatments were 21.3 and 5.1 µM, respectively. Then, we tested the inhibitory effect of the same concentrations of EPI-X4 and JM#21 peptides on CXCL12-induced migration of BCWM.1 cells (Figure S5B). While >50 µM of EPI-X4 peptide was required to significantly reduce the CXCL12-induced migration to 13 ± 1.3%, only 20 µM of JM#21 reduced it to 10 ± 2.7% compared with PBS treatment resulting in 19.3 ± 0.7% migration. Of note, 10 µM JM#21 and AMD3100 and 200 µM of EPI-X4 were found to be effective for suppressing the CXCL12-induced migration of BCWM.1 cells overexpressing CXCR4 isoform 1, which caused an unusual increase in specific migration up to 80% [31]. Taken together, the JM#21 derivative showed potency over the original EPI-X4 [31,36,39]. As we intended to prevent the enhanced CXCR4 activation upon mAbo treatment in wildtype BCWM.1 cells expressing endogenous CXCR4, we analyzed the combinatorial dose-dependent migration response of mAbo at concentrations ranging from 1 to 20 µg/mL and of JM#21 ranging from 5 to 100 µM in the presence of 60 nM CXCL12 (Figure 4A,B). In both the Loewe and HSA models, JM#21 antagonized the mAbo-induced enhanced migration response, specifically at higher dosages (Figure 4B). Unlike BCWM.1 cells, CXCL12-induced migration of DHL6 cells was readily diminished by very low doses (<20 µM) of JM#21 (Figure 4A,C). For further analysis, we selected the dosage combination of 20 µM JM#21 and 10 µg/mL mAbo, which corresponds to Loewe and HSA synergy scores in BCWM.1 cells at −24.4 ± 2.7 and −16.1 ± 2.6, respectively (Figure 4B). We evaluated a linear combinatorial effect of mAbo dosages in the presence and absence of 20 µM JM#21 at 60 nM CXCL12, revealing a significant retardation effect of JM#21 across various mAbo dosages, except for the very high dose of 20 µg/mL (Figure 4D). Similarly, we examined the linear combinatorial effect of mAbo dosages in the presence of 20 µM JM#21 on CXCL12-induced migration in MWCL-1 and OCI-Ly3 cells (Figure 4E,F). Notably, MWCL-1 and OCI-Ly3 cells are derived from the monoallelic del17p13 karyotype WM and activated B cell (ABC) type DLBCL, respectively [40,41]. These results show the general potency of JM#21 on other WM and DLBCL cell types.

### 2.6. CXCR4-Antagonizing Peptide Enhances CD19 mAbs-Induced ADCC

Next, we analyzed the interaction of mAbo and JM#21 in ADCC assay using combinatorial dose-dependent responses (Figure S5C,D). Both the HSA and Loewe models indicated an uniform combinatorial or additive effect, specifically at higher doses of JM#21 > 20 µM and mAbo > 10 µg/mL. Notably, scores between −10 and +10 suggest a primary combinatorial or an additive interaction [29,30]. Single-dose response data for mAbo and JM#21 are shown in Figure S5C. The specific synergy scores for our selected combination of 10 µg/mL mAbo and 20 µM JM#21 in Loewe and HSA models were 3.7 ± 1.5 and 5.2 ± 0.1, respectively (Figure S5D). To analyze the efficacy of EPI-X4 and its derivative JM#21 in CD19-dependent ADCC, we used CD19 and IgM KO BCWM.1 cells in the presence of mAbo and inactive Fc-silent mAbo FcS (Figure S5E). Expectedly, the combinatorial efficacies were observed only in the WT and IgM KO cells, but not the CD19 KO BCWM.1 cells. Similarly, inactive Fc-silent mAbo FcS showed no combinatorial efficacy in ADCC in the presence of EPI-X4 and JM#21 (Figure S5E).

We then compared the efficacy of 20 µM JM#21 in mAbo-induced ADCC and equivalent concentrations of EPI-X4 and AMD3100 (Figure S5F). While both JM#21 and EPI-X4 treatments improved the mAbo-induced ADCC and reduced the survival of BCWM.1 significantly compared with the no inhibitor control, AMD3100 treatment caused no significant difference (Figure S5F, upper panels). In contrast, all three CXCR4 antagonists caused no significant decrease in survival of DHL6 cells in mAbo-induced ADCC assays (Figure S5F, bottom panels). As a control, we also a performed mock ADCC coculture experiment with mAboFcS in the presence and absence of CXCR4 antagonists. As shown before, mAboFcS treatment did not cause any ADCC alone and survival loss; instead, it increased cell survival due to activation of CD19 signaling in the absence of NK ligation (Figure S5E,F and Figure 3A–C). However, in the presence of all the CXCR4 antagonists used, survival of mAboFcS-treated BCWM.1 cells was significantly decreased (Figure S5F). And, as shown before, DHL6 cells did not have any survival advantage upon mAboFcS treatment in the ADCC assay (Figure S5F and Figure 3D–F). Despite no added survival advantage, all CXCR4 antagonists except EPI-X4 caused significant decreases in survival in the mock ADCC assay in the presence of mAboFcS. These results demonstrate the differential effect of CXCR4 antagonists on CD19-mediated activation signals in lymphoma cells and warrant further improvement in the combinatorial efficacy of CD19 mAb-induced ADCC and enhanced migration. In particular, the peptide antagonist JM#21 effectively enhanced the mAbo-induced ADCC in both BCWM.1 and DHL6 cells as well as preventing the enhanced survival caused by mAboFcS due to lack of NK cell ligation.

**Figure 5:**
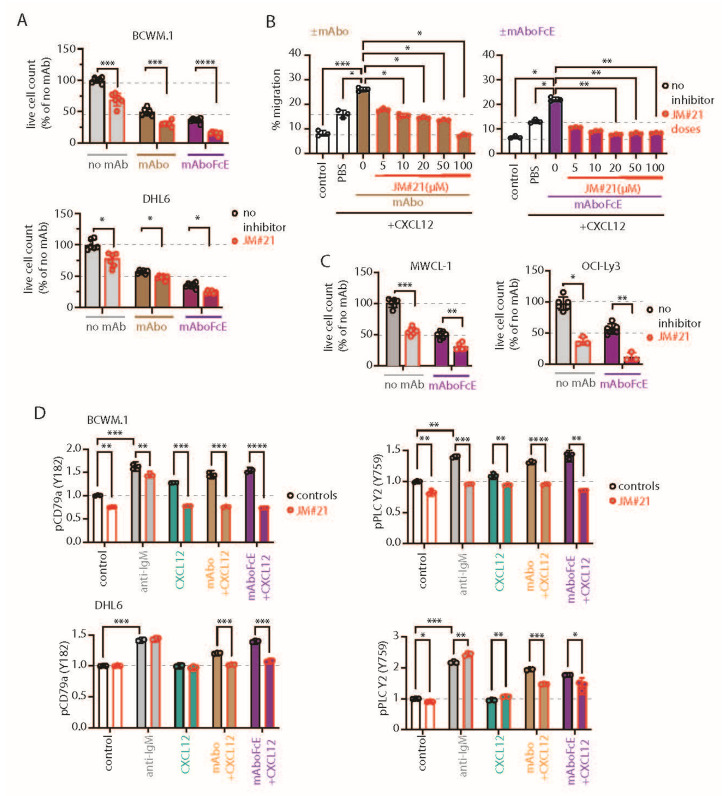
CXCR4 antagonizing peptides enhance CD19 mAbs-induced ADCC. (**A**) Quantification of live cell count of BCWM.1 (**upper panel**) and DHL6 (**lower panel**) cells in ADCC induced by mAbo (brown bars) and mAboFcE (purple bars) antibodies in the absence (black, open circles) and presence of 20 µM CXCR4 antagonist peptide JM#21 (red, filled circles). (**B**) Inhibitory dose response of JM#21 on enhanced CXCL12-induced migration of BCWM.1 cells in the presence of mAbo (**left panel**) and mAboFcE (**right panel**). (**C**) Same as (**B**) combinatorial effect of mAboFcE and JM#21 on MWCL-1 (**left panel**) and OCI-Ly3 (**right panel**) cells. (**D**) Effect of JM#21 on increased pCD79a (Y182) and pPLCγ2 (Y759) levels in BCWM.1 (**upper panels**) and DHL6 cells (**bottom panels**) in response to stimulants—anti-IgM, mAbo + CXCL12, and mAboFcE + CXCL12—for 5 min. Data in (**A**,**C**,**D**) were analyzed by Two-Way ANOVA followed by Dunnett’s multiple comparison, and data in (**B**) were analyzed by One-Way ANOVA followed by Dunn’s multiple comparison. * *p* < 0.05, ** *p* < 0.01, *** *p* < 0.001, **** *p* < 0.0001.

To this end, we tested the effect of JM#21 on the mAboFcE-induced ADCC on BCWM.1 and DHL6 cells and compared it with mAbo treatment (Figure 5A). The addition of 20 µM JM#21 significantly enhanced the efficacy of mAboFcE, causing decreases in the survival of BCWM.1 cells to 15.2 ± 2.8% from no inhibitor control 36.1 ± 3.8% (Figure 5A). In contrast, the survival in mAbo-induced ADCC was decreased only to 30.9 ± 4.1% from 40.7 ± 5.9%. The survival of DHL6 cells in ADCC assays with mAbo and mAboFcE in the presence and absence of 20 µM JM#21 was reduced to 47.7 ± 3.3% from control of 56.5 ± 2.7% and 24.2 ± 2.3% from 35.2 ± 4.3%, respectively. This result demonstrates the combinatorial efficacy of JM#21 and Fc-engineered CD19 mAb that surpasses the individual treatments. Next, we tested whether JM#21 prevents the CD19 mAb-induced enhancement of CXCL12-induced migration (Figure 5B). As shown before, mAbo treatment increased the CXCL12-induced migration of BCWM.1 cells (Figure 2C). This increased migration was inhibited by JM#21 in a dose-dependent manner significantly at doses > 10 µM (Figure 5B). Similarly, the enhanced CXCL12-induced migration caused by mAboFcE treatment was significantly blocked by JM#21 doses > 20 µM. In parallel, we tested mAboFcE’s combinatorial efficacy with EPI-X4 on CD19 KO BCWM.1 and DHL6 cells (Figure S6A,B). Expectedly, efficacy was seen only in WT and IgM KO cells, not in CD19 KO BCWM.1 (Figure S6A). Similarly, no additive effects were observed in CD19 KO DHL6 cells compared with their WT counterpart (Figure S6B). We also tested the efficacy of EPI-X4 to prevent the mAbo- and mAboFcE-induced increase in migration in BCWM.1 cells (Figure S6C). While a minimum of 50 µM EPI-X4 was required to significantly prevent mAbo-induced increased migration, above 10 µM, EPI-X4 effectively blocked the mAboFcE-induced enhanced migration. Thereafter, we tested the combinatorial efficacy of mAboFcE and JM#21 inducing ADCC in MWCL-1 and OCI-Ly3 cells (Figure 5C). The combination of mAboFcE and JM#21 was more effective for the OCI-Ly3 cells than the MWCL-1 cells. These data together showed JM#21 induced antagonism toward CD19-mediated migration and combinatorial efficacy in ADCC for other WM and DLBCL cells, namely, MWCL-1 and OCI-Ly3 (Figure 4E,F and Figure 5C). Together, these results show the efficacy of JM#21 over EPI-X4 against CD19 mAb-induced activation of CXCR4 signaling.

Next, we tested the effect of JM#21 on intracellular phosphorylation of BCWM.1 and DHL6 cells (Figure 5D and Figure S6D). As shown before, both BCR stimulation with anti-IgM and CXCR4 stimulation with CXCL12 treatment readily induced phosphorylation of pCD79a, pAKT, pERK, and pPLC-γ2 in BCWM.1 (Figure 2H and Figure S2H). In DHL6 cells, CXCL12 treatment in the presence of mAbo only induced pCD79a and pPLC-γ2. Therefore, we first analyzed the effect of mAboFcE in the presence of CXCL12 on the induction of pCD79a and pPLC-γ2 in both BCWM.1 and DHL6 cells, and then, combined with JM#21 treatments (Figure 5D). Congruent to the previous results using mAbo, the combination of mAboFcE and CXCL12 (mAboFcE+CXCL12) treatments caused increased pCD79a and pPLC-γ2 in both BCWM.1 and DHL6 cells compared with CXCL12-only treatments. In contrast, the mAboFcE + CXCL12 treatments induced pAKT and pERK in BCWM.1 cells only, similar to the mAbo + CXCL12 treatments (Figure S6D). To compare the effect of CXCR4 antagonist, we pretreated cells with 20 µM JM#21 similarly as in the ADCC assays and then stimulated them with anti-IgM, CXCL12, mAbo + CXCL12, and mAboFcE + CXCL12 (Figure 5D and Figure S6D). Congruent to the previous report, JM#21 treatment caused reduced basal phosphorylation of pAKT and pERK without any additional stimulation in BCWM.1 cells [31]. In addition, we found a decrease in basal pCD79a and pPLC-γ2 levels in BCWM.1 cells (Figure 5D). However, the basal phosphorylation for all four markers remained unchanged in DHL6 cells upon JM#21 treatments (Figure 5D and Figure S6D), suggesting a complete CXCR4 signaling independent survival of these cells, which is in resonance with the minimal CXCL12-induced migration (Figure 1J). Like reduced basal phosphorylation, JM#21 pretreatments reduced the anti-IgM and CXCL12-stimulated pCD79a, pAKT, pERK, and pPLC-γ2 induction in BCWM.1 cells only, not in DHL6 cells (Figure 5D and Figure S6D). In addition, JM#21 pretreatments inhibited the increased pCD79a, pAKT, pERK, and pPLC-γ2 in response to mAbo + CXCL12 and mAboFcE + CXCL12 stimulations in both cell types. As such, the synergistic increase in phosphorylation in DHL6 cells was less pronounced, and therefore, the effect of JM#21 pretreatments is minimal compared with BCWM.1 cells. Altogether these results show that in combination with JM#21, the efficacy of CD19 mAbs, including the modified FcR binding enhanced version (mAboFcE), was instantly increased by the enhanced ADCC and inhibition of the CD19-induced activation signal. Broadly, blocking CXCR4 with an optimized EPI-X4-derived peptide antagonist like JM#21 is a promising approach to augment the therapeutic effects of CD19 mAbs for the treatment of WM. To down-modulate the CD19 mAb-induced activation of the CXCR4 response, we then explored the combinatorial potential of CXCR4 inhibition to improve the mAb-induced ADCC. The endogenous peptide inhibitor of CXCR4 (EPI-X4) specifically antagonizes CXCR4 and is, therefore, a promising candidate for drug development for the treatment of CXCR4-dependent diseases [35]. Optimized EPI-X4 derivatives reduced the tumor burden in different cancer models, and specifically, the survival of the WM cell line in immunocompromised mouse recipients [31,36,37]. We here tested the original EPI-X4 and an optimized version named EPI-X4 JM#21 (herein referred to as JM#21), which showed 1000-fold increased antagonistic activity compared with the WT peptide [37]. In addition, we included the small-molecule CXCR4 antagonist AMD3100.

## 3. Discussion

In this study, we investigated the role of CD19, a crucial co-receptor for B-cell receptor (BCR) signaling, in the context of B-cell lymphomas, including aggressive forms like diffuse large B-cell lymphoma (DLBCL) and indolent forms like Waldenström Macroglobulinemia (WM). Our study highlights the essential role of CD19 in the survival, proliferation, and CXCL12-induced migration of lymphoma cells. In parallel, treatment with certain anti-CD19 monoclonal antibodies (mAbs) effectively enhances the CXCL12-induced migration of WM cells. The generation of CD19 KO BCWM.1 and DHL6 cell lines provided a crucial model to elucidate the role of CD19 in WM and GCB-DLBCL, respectively. Both cells were marked by deficiencies in cell growth and CXCL12-driven migration upon CD19 loss (Figure 1 and Figure S1). Altogether, the CD19-regulated pathways are critical for both growth and maintenance of B-cell lymphomas as well as chemotaxis, a crucial step toward dissemination.

We found that CD19 KO cells were significantly outcompeted by wildtype (WT) cells in coculture assays (Figure 1A), indicating the necessity of CD19 for maintaining cell viability under competitive conditions. This was further evidenced by the reduced number and size of colonies formed by CD19 KO cells in CFC assays (Figure 1B–E). Apart from growth retardation, the CD19 KO cells showed no CXCL12-induced migration, highlighting the critical role of CD19 in maintaining B-cell lymphoma viability and mobility (Figure 1F–H). Additionally, treatment with anti-CD19 mAbs increased migration. However, the increase was largely dependent on the mAbs’ binding specificity and clonotypes, underscoring the complex impacts of targeting CD19, which can potentially promote lymphoma cell growth under certain conditions.

Targeting CD19 with mAbs has become a focal point in personalized medicine due to its ubiquitous expression in all B lineage cells, including neoplastic and autoimmune B cells [42]. However, the enhanced migration capabilities of lymphoma cells toward CXCL12 indicate that CD19’s signaling interaction with CXCR4 is a crucial mechanism in lymphoma cell chemotaxis. Furthermore, our findings underscored the variable efficacy of different CD19 mAb clones. While some clones significantly enhanced CXCL12-induced migration, others had minimal effects, highlighting the importance of selecting the appropriate therapeutic clone to target specific lymphoma subtypes [6]. The Fc silent (FcS) versions of these mAbs maintained similar functionalities, indicating that the effects were predominantly mediated through CD19 signaling rather than secondary Fc receptor engagement on the same B cell surface. Contrastingly, experimental results showed that a failure to ligate NK cells, as demonstrated by FcS mutations, enhances CD19-mediated pathways and confers a survival advantage to lymphoma cells. Thus, the availability of NK cells and the type of anti-CD19 mAb used together determine the treatment outcome. To our knowledge, such direct comparative studies elucidating the differences in CD19 mAb clones have not been conducted. Together, our findings support the notion that the optimal schedule and type of CD19 mAbs in therapy has yet to be determined [7].

We show that CD19 mAbs significantly enhance cytotoxicity, particularly when combined with CXCR4 antagonists. This combination therapy not only inhibited the survival and proliferation of lymphoma cells by intervening in the CD19–CXCR4 signaling crosstalk, but also enhanced their susceptibility to ADCC. The overexpression of CXCR4 and introduction of activating mutations were associated with poor prognosis and an impaired response to rituximab [43]. The process facilitates lymphoma cell migration toward CXCL12-producing stromal cells and promotes severe bone marrow infiltration. Also, the dissemination patterns of most B-cell malignancies reflect the fundamental principles of lymphocyte homing to secondary lymphoid organs and the restricted tissue-specific egress, indicating a reiterated chemokine receptor signaling. This signaling overdrive suggests a mechanism by which lymphoma cells evade treatment, particularly noted in B-cell malignancies like WM, acute lymphoblastic leukemia, and multiple myeloma [36,44,45]. Under these conditions, lymphoma cells exploit the enhanced chemokine receptor signaling to hide within bone marrow niches and stay away from the grasp of infused mAbs and chemotherapy. While our study primarily focused on DLBCL and WM, the implications of targeting the CD19–CXCR4 signaling axis extend beyond these lymphoma subtypes. CXCR4 overexpression and constitutive activation are well-documented features across various mature B-cell malignancies, including chronic lymphocytic leukemia (CLL), mantle cell lymphoma (MCL), and certain subtypes of follicular lymphoma (FL). Notably, CXCR4 facilitates bone marrow retention and extramedullary dissemination in these malignancies, often correlating with adverse clinical outcomes and therapy resistance. CD19-targeted immunotherapies such as mAbs and CAR-T cells have already demonstrated clinical success across multiple B-cell neoplasms; however, resistance mechanisms driven by CXCR4-mediated microenvironmental protection may similarly undermine these therapeutic approaches. Our data suggest that inhibiting CXCR4 signaling with JM#21 can synergize with CD19 mAbs to enhance lymphoma cell eradication by reducing tumor cells’ dissemination and improving ADCC. This combination approach holds promise not only for DLBCL and WM but also for other B-cell malignancies wherein CXCR4 signaling contributes to disease progression and therapy resistance. Future studies validating this strategy in diverse B-cell malignancies could further broaden the clinical application of CXCR4-targeted adjuncts in CD19-based immunotherapy.

Here we used JM#21, a peptide antagonist derivative of the naturally occurring CXCR4 peptide blocker EPI-X4 [35,37], which inhibited the growth of WM in previous studies [31]. Similarly, a fatty acid modified derived from EPI-X4, namely JM#170, inhibited the growth of B-cell acute lymphoblastic leukemia (B-ALL) [36]. In this study, we demonstrated that the CXCR4 antagonist EPI-X4 and its optimized derivative, JM#21, effectively enhance the therapeutic effects of CD19-targeted immunotherapy in B-cell lymphomas, particularly in DLBCL and WM. Both the parental peptide and the optimized derivative disrupted CXCL12-mediated signaling, which plays a key role in lymphoma cell migration and survival, especially in CXCR4-expressing malignant B cells. Although EPI-X4 and its optimized derivative JM#21 have been extensively studied for their selectivity toward CXCR4, off-target effects cannot be fully excluded [35,46]. While a comprehensive receptor-wide screening has not been conducted for JM#21, current data suggest that its off-target activity is limited. Recently, JM#21 has been found to interact with atypical chemokine receptor 3 (ACKR3), competing with CXCL12 at its binding site (unpublished data, Albers et al.), though the functional relevance of this interaction remains to be clarified [47]. In contrast, JM#21 suppresses CXCR4-tropic HIV-1 infection more efficiently than the clinically approved small-molecule CXCR4 antagonist AMD3100 [37]. In WM cells, control peptides lacking CXCR4-binding capacity (CTL) failed to inhibit CXCL12-induced migration or rescue survival after xenografting, further supporting the CXCR4-specific action of JM#21 [31]. Binding site analyses and molecular dynamic simulations indicate that JM#21 interacts with the CXCR4 minor pocket similarly to EPI-X4, thereby preserving specificity while exhibiting additional inhibitory effects, including anti-inflammatory properties and the suppression of interferon signaling [46,48]. In contrast, the FDA-approved CXCR4 antagonist AMD3100 primarily interacts with the major CXCR4 binding pocket, showing no effect on anti-inflammatory or interferon pathways but effectively mobilizing lymphoma cells from bone marrow niches into the periphery [48,49]. Notably, recent studies demonstrated that EPI-X4 derivatives induce cytotoxicity in B-ALL, whereas AMD3100 fails to do so [36]. Furthermore, different CXCR4 antagonists can induce distinct receptor dimerization patterns, favoring minor pocket-binding molecules like EPI-X4 derivatives [50]. Thus, JM#21 and related peptides exhibit unique functional properties compared with AMD3100. By inhibiting CXCR4 signaling, JM#21 significantly enhances ADCC, demonstrating a robust combinatorial effect (Figure 4B and Figure S5D). Simultaneously, the JM#21 antagonized the CXCR4 and prevented CXCL12-induced migration upon CD19 mAb treatment. Our findings underscore the potential of combining CD19-targeted immunotherapy with CXCR4 antagonists to overcome the resistance mechanisms often associated with CXCL12–CXCR4 signaling in lymphoma cells [15,16,43]. This approach not only enhances mAb efficacy but may also limit the dissemination of malignant B cells, which rely on CXCR4 for homing to protective niches in the bone marrow and lymphoid tissues [6,12]. These results support further investigation into EPI-X4 derivatives with increased stability [39,51] as adjuncts to CD19-targeted therapies, offering a promising strategy for enhancing clinical outcomes in CXCR4-dependent lymphomas.

In conclusion, our results illustrate the complex interplay between CD19 and CXCR4 in lymphoma pathophysiology and therapeutic response. The insights gained from this study advocate for the continued development of targeted therapies that leverage the synergistic effects of CD19 and CXCR4 inhibition. Future research should focus on evaluating the efficacy of these combined therapies in diverse lymphoma settings, aiming to optimize treatment regimens and improve outcomes in B-cell lymphoma treatment.

## 4. Methods and Materials

### 4.1. Antibodies and Other FACS Reagents

For FACS-based receptor expression and intracellular phospho-flowcytometry analyses of different gene knockout (KO) cells, the following anti-human antibodies were used: anti-IgM Alexa Fluor 647 (Cat. No.314536), anti-IgM-BV605 (Cat. No. 314524), anti-IgD-APC (Cat. No. 348222) and anti-CXCR4-BV421 (Cat. No. 306518) from Biolegend, San Diego, CA, USA; anti-CD19-BV786 (Cat. No. 563325), pERK(pT202/pY204)-AF488 (Cat No. 612592), pAKT-BV421(pS473) (Cat. No. 562599) and pPLC-γ2 (pY759)-PE (Cat. No. 558490) form BD Biosciences, Heidelberg, Germany; pCD79a(Y182) AF647 (Cat. No. 297425), and pAKT(S473)-PE-Cy7 (Cat. No. 881065) form Cell Signaling Technology, Danvers, MA, USA. For live cell counting: Sytox™ Blue Live-Dead Cell Stain (Cat No. S34857) and AccuCheck Counting Bead (Cat. No. PCB100) were obtained from Invitrogen, Dreieich, Germany.

### 4.2. Cell Culture

WM cell lines BCWM.1 and MWCL-1 were a kind gift from Steven P. Treon [40,52]. Germinal center B cell (GCB) type DLBCL cell line SU-DHL-6 (called DHL6) and activated B cell (ABC) type cell line OCI-Ly3 were obtained from German Collection of Microorganisms and Cell Cultures (DSMZ Repository ID ACC-572 and ACC-761). All cell lines were cultured in complete RPMI medium (Invitrogen) containing 10% FBS (type: standard; Pan Biotech, Aidenbach, Germany), 10 U/mL penicillin and 100 µg/mL streptomycin, 2 mM L-Alanyl-L-glutamine solution (stable glutamine), 1 mM sodium pyruvate, 5 µM β-mercaptoethanol (all from Invitrogen) and 10 mM HEPES (Sigma-Aldrich, Hamburg, Germany) at 37 °C in humidified 5% CO_2_ incubator. For recombinant antibody production, HEK293T were maintained in complete Iscove’s medium (Sigma-Aldrich) containing 5% FBS (type: standard, Pan Biotech), penicillin/streptomycin, glutamine, sodium pyruvate, and 50 mM β-mercaptoethanol (Gibco, Dreieich, Germany) at 37 °C in humidified 7.5% CO_2_ incubator. For retrovirus and lentivirus packaging, HEK293T derivative Phoenix-Eco and Lenti-X™ 293T (Takara Bioscience, Kyoto, Japan) cells were maintained under similar conditions.

### 4.3. Immunochemical, Cytokines, and Inhibitors

For stimulations following immunochemical and cytokines, we used: anti-IgM goat polyclonal (Cat. No. 2020-01, Southern Biotech, Birmingham, AL, USA), rhCXCL12 (Cat. No. 300-28A, PeproTech, Hamburg, Germany), rhIL-2 (Cat. No. 11340025, ImmunoTools, Friesoythe, Germany). Preparations of CXCR antagonists EPI-X4 and its derivative JM#21 and their usage on WM cells were previously described [31,37]. In brief, lyophilized EPI-X4 (Mol. Wt. 1832) and JM#21 (Mol. Wt. 1458) peptides were dissolved in H_2_O, stored frozen as 5 mM stocks, and further diluted in phosphate-buffered saline (PBS) or cell culture media as necessary for usage. Similarly, the AMD3100 octahydrochloride (Cat. No. S3013, Selleckchem, Cologne, Germany) was also dissolved in H_2_O and stored frozen as 5 mM stock.

### 4.4. CRISPR-Cas9 Plasmids Generation

The lentiviral Cas9 expression blasticidin resistance (Bsd^R^) plasmid pL Cas9-mCat-1 Bsd^R^ was generated by constructing N-terminal FLAG-tagged spCas9 (Addgene #48138) followed by p2a self-cleavage peptide linked to murine cationic amino acid transporter-1, mCat-1 (Addgene #17224) for γ-ecotropic retroviral susceptibility. The cassette, followed by Bsd^R^ gene under SV40 promoter, was then inserted into lentiviral vector pCDH_MSCV (System Bioscience, Palo Alto, CA, USA). The designing of the guide RNA (sgRNA) for CRISPR-Cas9 was performed by using CrispR Tool in Geneious Prime^®^ software (https://www.geneious.com/) by selecting protospacer adjacent motif (PAM) option [53]. Two sets of highest-scoring sgRNAs along with extensions needed for cloning in BbsI sites were synthesized from Eurofin Genomics as complementary paired oligos, annealed, and cloned into the BbsI sites of pR_U6sgRNA_mRFP-1 (Addgene plasmid *#*112914) or pR_U6sgRNA_eFGP(Addgene plasmid *#*116926) for IgM and CD19, respectively. Finally, the most effective target sequences were determined by frequency of deleted cells in FACS analyses post transduction. Selected target sgRNAs were IGHM: 5′ CCCGTCGGATACGAGCAGCGTGG 3′and CD19: 5′ GGTCTC-GGGAGTCCCCGCTT 3′.

### 4.5. Lentiviral and Retroviral Transductions

For lentivirus preparation, Lenti-X™ 293T cells were transfected with pL Cas9-mCat-1 Bsd^R^ plasmid together with helper plasmids pMD2.G (Addgene #12259) and pxPAX2 (Addgene #12260), mixed at a ratio of 9:7:4 using PolyFect (Qiagen, Venlo, The Netherlands) reagent. The produced virus particles were concentrated from the culture supernatant after 72 h using Lenti-X™ Concentrator (Takara Bioscience) according to manufacturer’s instructions, and the concentrated virus medium (VCM) aliquots were stored at −80 °C until use. For transduction, RetroNectin^®^ (Takara Bioscience)-coated 6-well plates were added with 1 mL of VCM supplemented with 8 µg/mL polybrene centrifuged at 1800 rpm for 3 h at 32–37 °C. After removing VCM, the 1 × 10^6^ lymphoma cells were added to each well containing 2 mL of complete RPMI, and the plates were centrifuged at 1200 rpm for 30 min at 32–37 °C followed by incubation at 37 °C and 5% CO_2_. After 2 days of incubation, all the adherent and non-adherent cells were collected, washed, and replated at an estimated seeding density of 100–250 K cells/mL and cultured in larger vessels or flasks. On day 6, cells were added with 20 µg/mL of Blasticidin and continued in culture with fresh Blasticidin every 2 days for 8 days. Positively selected cells were tested for Cas9 and mCat-1 (Cas9Eco cells) expression by performing FACS staining with anti-mouse mCat-1 APC (Cat. No. 150505, Biolegend) and anti-FLAG Alexa Fluor 647 (cat no. NB600-344AF647, Bio-techne, Minneapolis, MN, USA) intracellular labeling using FIX&PERM^®^ (NordicMUBio, Susteren, The Netherlands) cell fixation and permeabilization kit according to manufacturer’s instructions. All procedures related to lentiviral packaging and transductions until complete removal of viral particles were carried out in BSL-2 environment and approved by institutional genetic engineering and GMO regulatory.

Retroviral transduction of the CD19 and IgM targeting sgRNA plasmids was performed as previously described [54,55]. In brief, HEK293T or Phoenix-Eco cells were transfected with BCR-encoding retroviral plasmids together with ecotropic packaging helper plasmid using the GeneJuice Transfection Reagent (Millipore, Darmstadt, Germany) as recommended by the manufacturer’s protocol. Culture supernatants containing the matured retroviral particles were collected 72 h after transfection and used for the subsequent transduction of Cas9Eco lymphoma cells by the spin-infection method in the presence of 5 µg/mL polybrene (Millipore). The transduction efficiency was evaluated four to five days after inoculation by measuring the percentage of the GFP- or mRF1-positive cells and respective targeted receptor.

### 4.6. Generating CD19 KO Clones

For the generation of single-cell CD19 KO clones, GFP-positive cells were sorted as single cells in 96-well U-bottom suspension plates containing 75 µL of complete RPMI medium in each well and maintained in a 37 °C, 5% CO_2_ incubator. After 15 days, 75 µL of additional medium was added, and the growing clones were identified by changing the color of the media or could be observed under the microscope. For sequencing, genomic DNA was isolated from each grown clone, and around 500 bp regions encompassing the sgRNA target site were PCR amplified and sequenced. The primers used for genotyping and sequencing were IGHMCµ1_F 5′ CCCCAGCAGCCTTGGACAAAGACC 3′, IGHMCµ1_R 5′ GCTGGACTTTGCACACCACGTGTTCG 3′, CD19Intron1_F01 5′GTGTGCAGCGTAAAATTCAGGAAAGGGTTGGAAGG3′ and CD19Exon2_R03 5′AGTCGAGATACATGACTGTCCAGCCAGGCTGCCAG 3′.

### 4.7. Colony-Forming Ability (CFA) Assay

For each KO clone, 2000 cells in 100 µL of serum-free Isocove’s medium were added with 2 mL prewarmed aliquots of Methocult™ 4236 (Stemcell Technologies, Vancouver, BC, Canada) media supplemented with 25 µL of 100× penicillin/streptomycin (Gibco) and 25 µL 1 M HEPES solution (Sigma). Total volume was adjusted with serum-free Iscove’s media up to 2.5 mL and vortexed vigorously for 20 s. Upon settling the medium for 5 min, triplicates of 500 µL mixture were distributed in a 12-well plate (Corning, Berlin, Germany) using a 16-gauge blunt-end needle. To prevent evaporation and retain humidity, sterile water was added in between the wells and incubated at 37 °C, 5% CO_2_ for 8–10 days. The CFC plates were scanned in Incucyte S3 Live Cell imager (Sartorius, Göttingen, Germany) at 10× magnification with an overlapping 7 × 7 image grid. The images were stitched, and the colonies were scored according to their number and size in Fiji (ImageJ2 version 2.14.0/1.54f).

### 4.8. ELISA

The day before the ELISA assay, 96-well plates were coated with 10 µg/mL goat polyclonal anti-human-IgM antibody (Cat. No. 2020-01, Southern Biotech) or goat polyclonal anti-human-IgG antibody (Cat. No. 2040-01, Southern Biotech) and incubated overnight at 4 °C. The next day, plates were washed with PBS containing 0.5% Tween-20, blocked with 1% BSA containing wash buffer for 1 h at 37 °C. Afterwards, serial dilutions of culture supernatant for IgM secretion or purified mAbo were added. For standard curve, 1:3 serial dilutions starting from 1 µg/mL of human IgM or IgG standard were added. Plates were incubated for 1 h at 37 °C, washed, and then added with 1:3000 dilution of Alkaline phosphatase (AP) conjugated IgM (Cat. No. 9020-04, Southern Biotech) or IgG (Cat. No. 2020-04, Southern Biotech) detection antibodies for 1 h. ODs were then measured at 405 nm in a multiplate reader 30 min after adding the AP substrate solution, followed by stopping with 1:3 volume of 3 M NaOH.

### 4.9. Competitive Survival Assay

For the suspension cell growth assay, individual single cell clones of knockout (KO) cells were cocultured with wildtype (WT) cells at an initial starting ratio of 1:1 in a 96-well plate, each well receiving a total of 100 K cells. The growth was determined by counting the Sytox™ Blue negative live cells with reference to AccuCheck, counting beads every 24 h interval from day 0 to day 4 using FACS-based assay. The CD19 KO and IgM KO cells were identified by reporter GFP and mRFP1 expressions, respectively.

### 4.10. Generation of Recombinant Anti-CD19 Monoclonal Antibodies

To generate CD19 mAbs, functional VJ and VDJ sequences derived from in-house subcloned anti-CD19 hybridoma, derived from CAT.131E10, were cloned into human Igκ light chain and IgG1 heavy chain, respectively. A self-cleaving p2a peptide linked the Igκ and IgG1 chains to produce a single-chain recombinant humanized anti-CD19 IgG (called mAbo) construct, cloned in modified pRVL IgG1 (Addgene #104583) expression vector with episomal amplification system [34]. The Fc receptor non-binder or Fc silent (FcS) and Fc receptor binding enhanced (FcE) versions were generated by introducing point mutations, as described before [33,34]. Two other humanized anti-CD19 mAbs, named mAb1 and mAb2 (Cat. No. Ab01511-10.0 and Ab00823-10.0), and their respective FcS versions, named mAb1FcS and mAb2FcS (Cat. No. Ab01511-10.3 and Ab00823-10.3), were purchased from Absolute Antibody, UK for research use only. Reportedly, both mAb1 and mAb2 are either in therapeutic usage or preclinical studies for directly targeting B-cell lymphoma or toxin delivery (Absolute Antibody, UK). For the expression of mAbo antibodies, 12 × 10^6^ HEK293T cells were seeded on a 15 cm adherent culture dish a day before transfection. Approximately 80 µg of plasmids were transfected using PEI Max^®^ (Polysciences, Warrington, PA, USA) and cultured in 20 mL complete media [34]. After 48 h, additional 20 mL serum-free medium was added to each plate. The antibody-containing supernatant was collected at day 5 of transfection, filtered, and concentrated with VivaSpin^®^ 100 KDa ultrafiltration units (Sartorius). For purification, HiTrap Protein G HP 1 mL columns were used in an ÄKTApure chromatography system (Cytiva, Uppsala, Sweden). Antibodies were eluted in 500 µL fractions with 100 mM glycine–HCl, pH 2.7, and immediately neutralized with equal volume of 1 M Tris buffer, pH 11.0. Neutralized antibody preparations were buffer exchanged with spin desalting columns in PBS. The numbers of antibodies and their purity were measured by ELISA and SDS-PAGE analyses of the fractions.

### 4.11. NK Cell Preparation and ADCC Assay

To isolate natural killer (NK) cells, peripheral blood mononuclear cells (PBMCs) were purified from buffy coats by Ficoll (Cytiva) density gradient centrifugation. Subsequently, the cells were labeled with CD56 microbeads (Cat. No. 130-097-42, Miltenyi Biotec, Bergisch Gladbach, Germany) and incubated for 15 min. Next, the labeled PBMCs cells were washed in MACs buffer, loaded into MACS Columns, and placed in the magnetic separator. The columns were washed three times for depleting unlabeled cells. Magnetically labeled CD56-positive NK cells were retained and eluted as the positively selected cell fraction.

Isolated NK cells were first labeled with 0.5 µM of CellTrace™ Far Red dye (Invitrogen) according to manufacturer’s protocol. To activate, labeled NK cells were stimulated with 10 ng/mL of rhIL-2 overnight (18–22 h) in complete RPMI medium containing 10% FCS. On the day of the experiment, frequencies of live–dead fractions were determined for both activated NK cells and lymphoma cells and resuspended at a concentration of 1 × 10^6^ cells/mL in serum-free RPMI. To combine KO and WT, cells were mixed at equal ratios and kept at final concentration of 1 × 10^6^ cells/mL. For ADCC, lymphoma cells were mixed with activated NK cells at a ratio of 1:4, mixed and added with 5 µg/mL of mAbs. For CXCR4 antagonist treatment, lymphoma cells were preincubated with 20 µM of peptide inhibitor and maintained in the same condition during the ADCC assay. After 4 h of coculturing, cells were analyzed by FACS after adding AccuCheck counting beads and 2 µM Sytox™ Blue (Thermo Fisher, Waltham, MA, USA).

### 4.12. Migration Assay (Chemotaxis Assay)

Lymphoma cells were resuspended in serum-free RPMI media at 2 × 10^6^/mL. Then, 50 µL of cells was loaded in the upper chamber of an 8.0 µm pore size HTS Transwell^®^-96 well plate (Corning). The lower chamber was filled with 150 µL of serum-free media with or without rhCXCL12 (60 nM) and anti-human CD19 (5–10 µg/mL). Cells that migrated into the lower chamber were harvested after 4 h of incubation at 37 °C, 5% CO_2_, and counted by using CellTiter-Glo^®^ 2.0 Assay (Promega, Madison, WI, USA) according to manufacturer’s instructions.

### 4.13. Phospho-Flow Assay

Lymphoma cells were resuspended in 1% FBS Iscove’s media at 20 × 10^6^ cells/mL, and the aliquots 50–75 µL (1–1.5 × 10^6^ cells) were added in each 1.5 mL Eppendorf tube. Cells were incubated at 37 °C for 20–30 min in a tabletop shaker before the addition of stimuli. Meanwhile stimulating antibodies were prepared by dissolving in 1% Iscove’s media at 2× dissolved final concentration in 50–75 µL volume. The prepared stimulating antibodies were added to the incubated samples according to the experimental design time points as 0’, 5’, 10’, and 20’. After each time point, 0.5% Sodium Azide prepared in PBS (ice cold) was added and washed by centrifuge at 500 g for 5 min at 4 °C. The cells were fixed by adding 4% Paraformaldehyde (PFA) incubated at RT for 20 min or directly by 1× prewarmed BD Phosflow™ Lyse/Fix Buffer (Cat. No. 558049, BD Bioscience) for 20 min at RT. For staining of cells, the antibodies were dissolved in a 1× permeabilization buffer (GAS-002B-1, NordicMUBio) added to the cells and incubated for 15–30 min at room temperature. Finally, cells were washed with 0.5% Saponin prepared in PBS. Cells were recorded in FACS Buffer (3% FBS, 0.1% Sodium Azide in PBS) at BD Fortessa (BD Bioscience).

### 4.14. Data Analysis

Statistical analysis was performed using GraphPad Prism 10.4 software. Wherever possible, specific statistical tests and numbers of replicates are mentioned in the figure legends. All FACS data were plotted and analyzed in FlowLogic 8.4 software. All image data were visualized and analyzed by Fiji (ImageJ2 version 2.14.0/1.54f) software.

## Data Availability

All data supporting the findings of this study are available within the article and its supplementary information files.

## Supplementary Materials

The following supporting information can be downloaded at: https://www.mdpi.com/article/10.3390/ijms26052024/s1.
