# Supplementary material for: CXCR4 Inhibition Enhances the Efficacy of CD19 Monoclonal Antibody-Mediated Extermination of B-Cell Lymphoma
Source: Int J Mol Sci. 2025 Feb 26;26(5):2024. doi: 10.3390/ijms26052024 (PMC11899823; doi:10.3390/ijms26052024)

# Supplement Figure S1

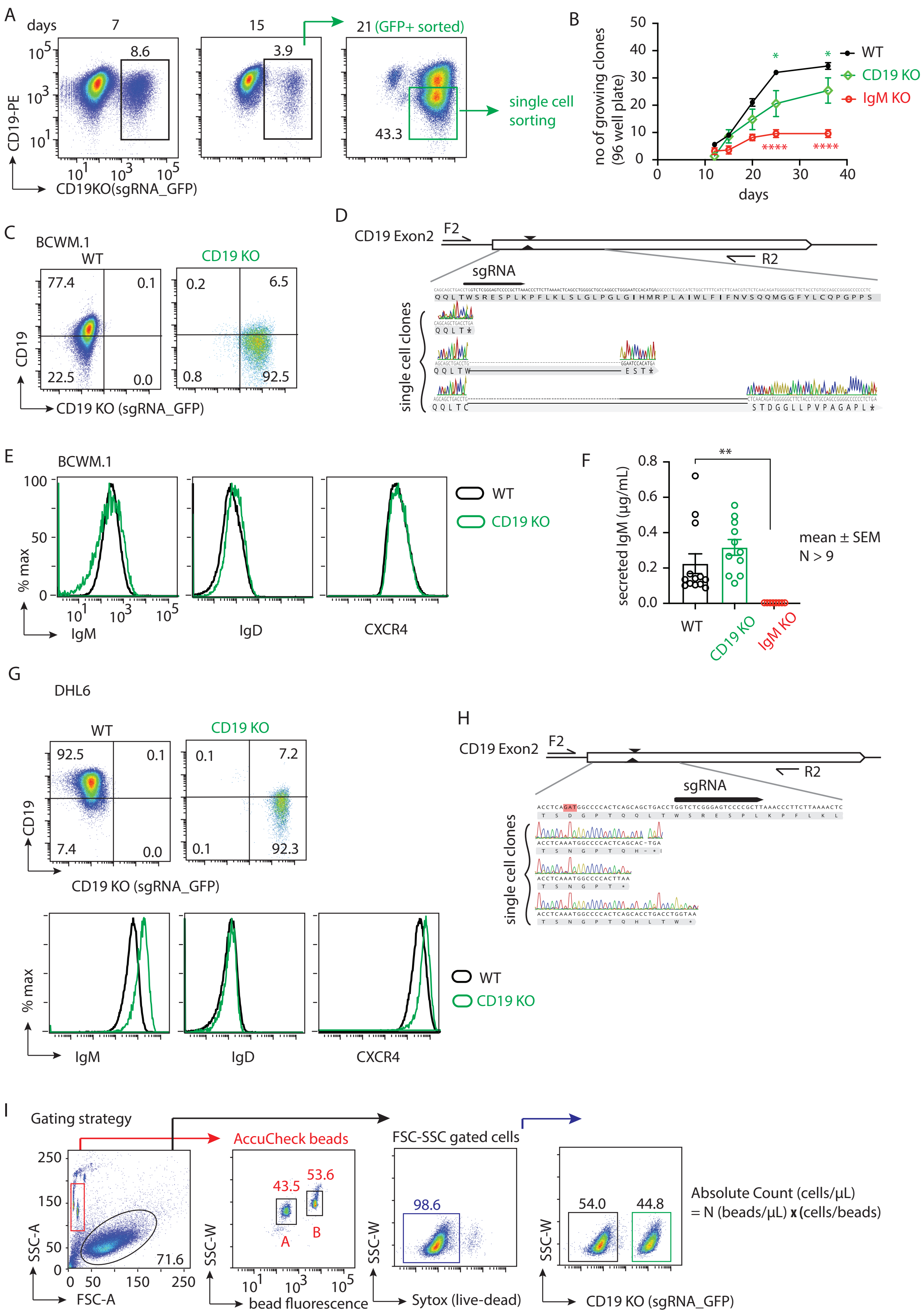

## Supplement Figure S2

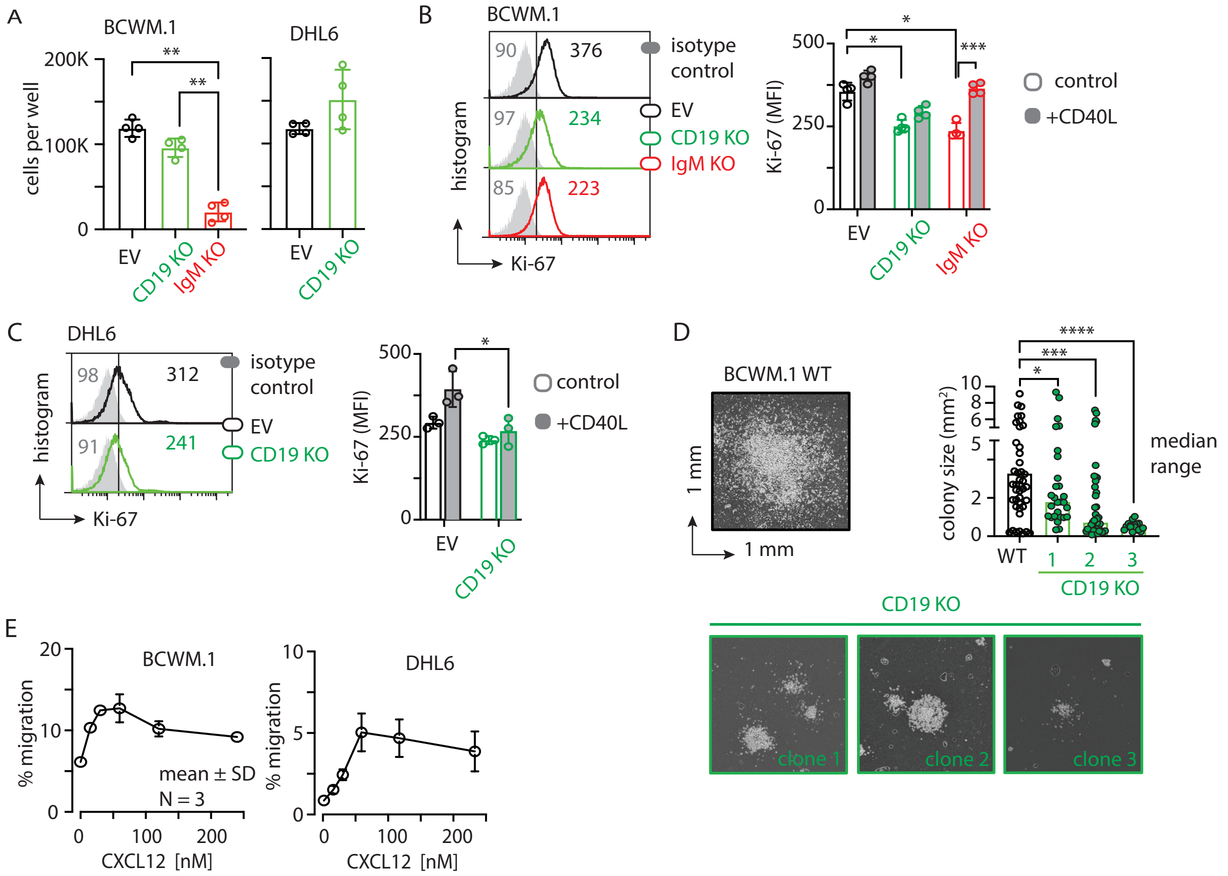

Supplement Figure S3

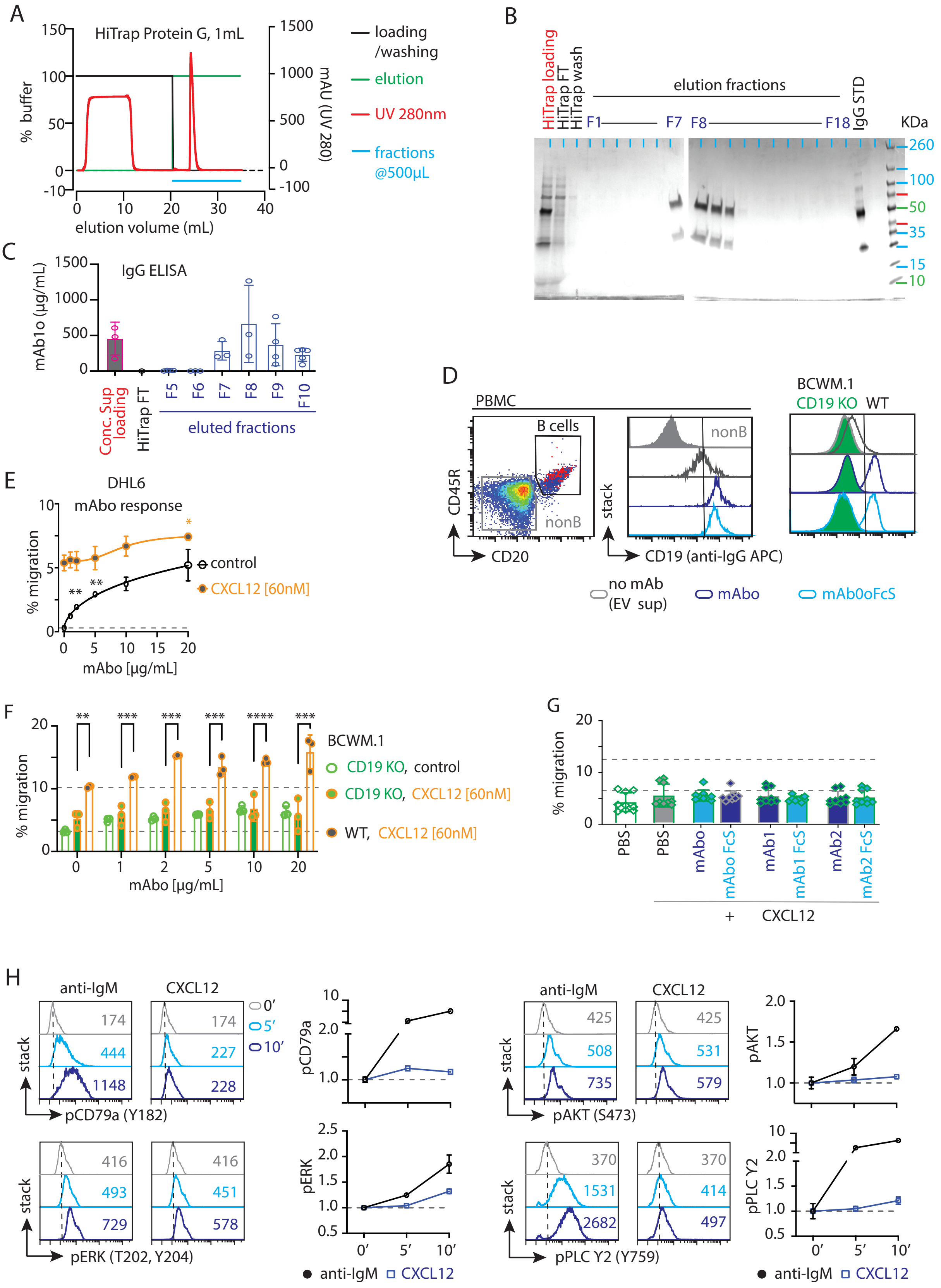

## Supplement Figure S4

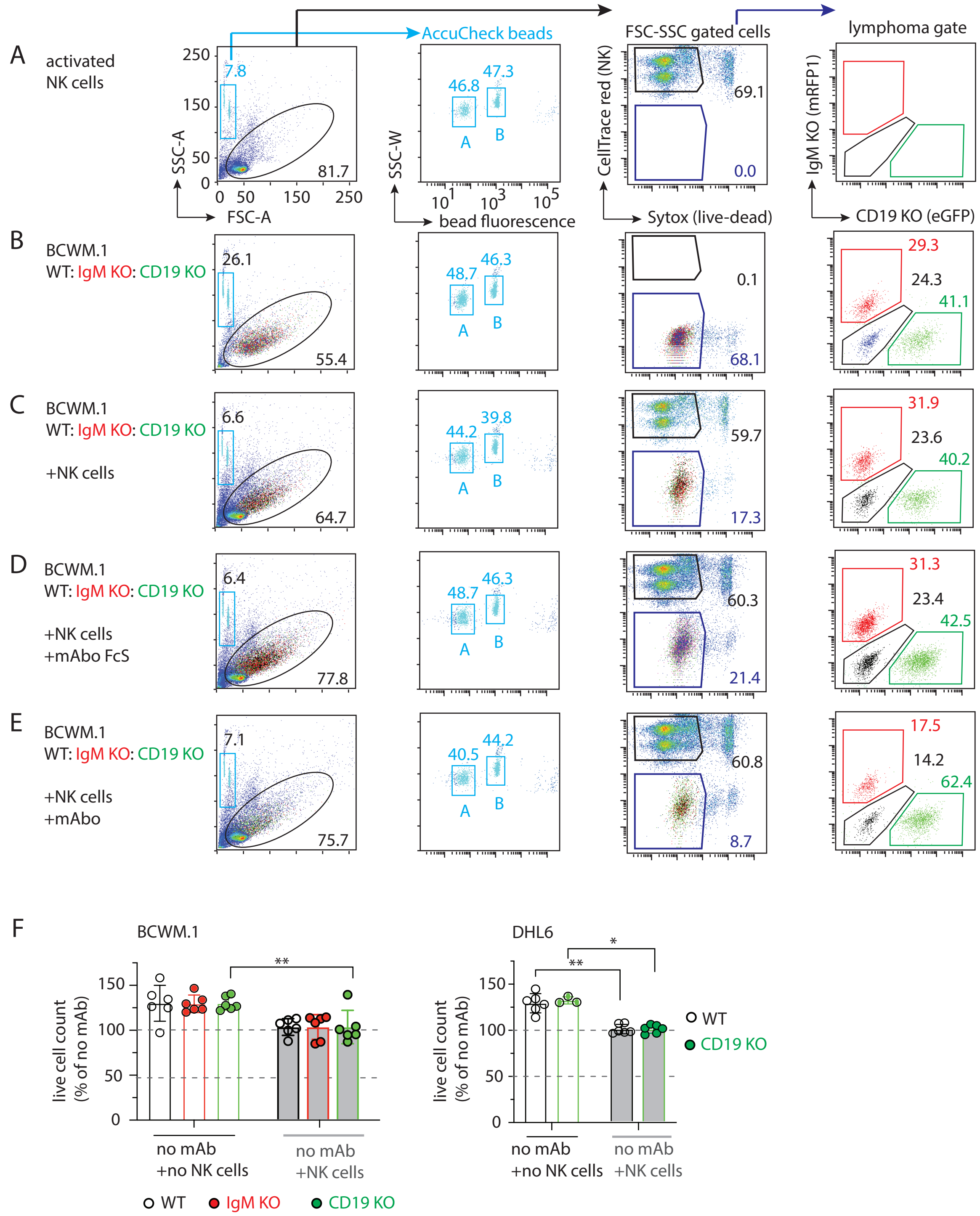

Supplement Figure S5

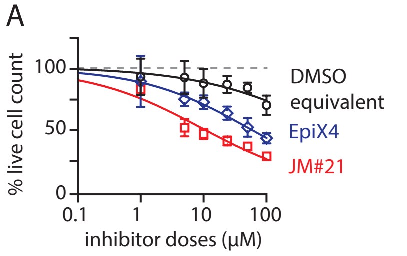


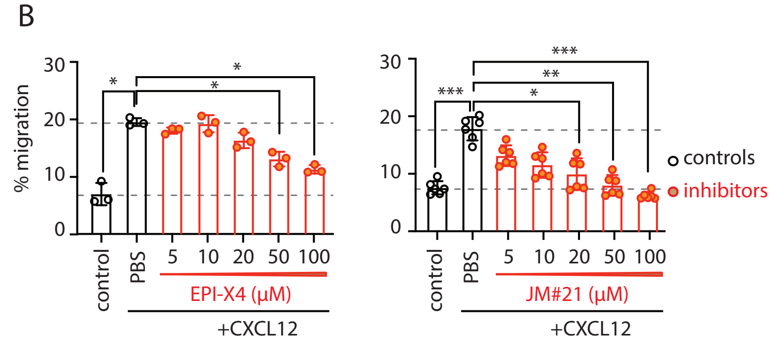


| Best-fit values | EpiX4 | JM#21 |
|---|---|---|
| Top | = 100.0 | = 100.0 |
| Baseline | Unstable | Unstable |
| Bottom | = 5.000 | = 5.000 |
| AbsoluteIC50 | 21.13 | 5.083 |
| HillSlope | -0.5066 | -0.4804 |

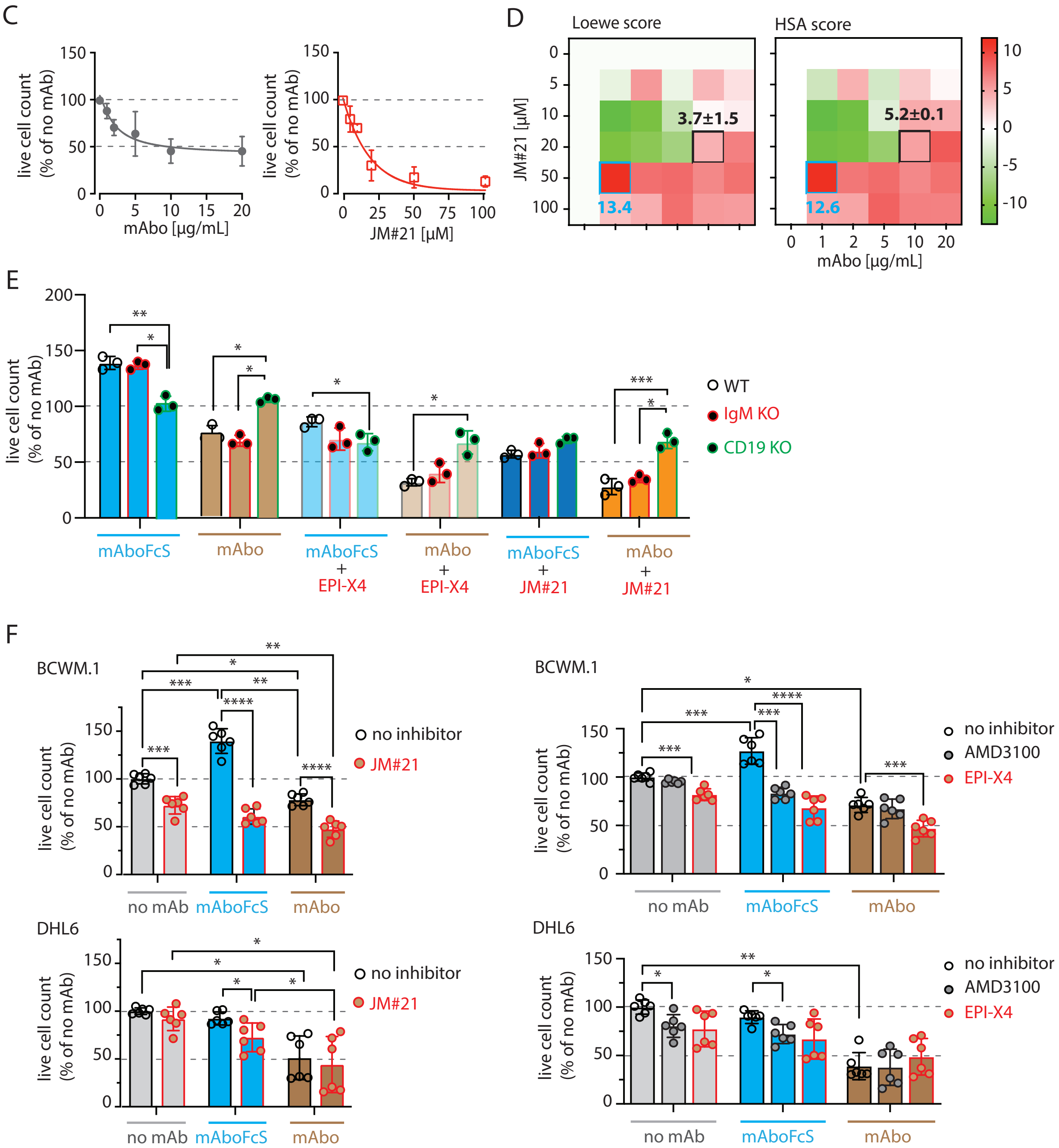

Supplement Figure S6

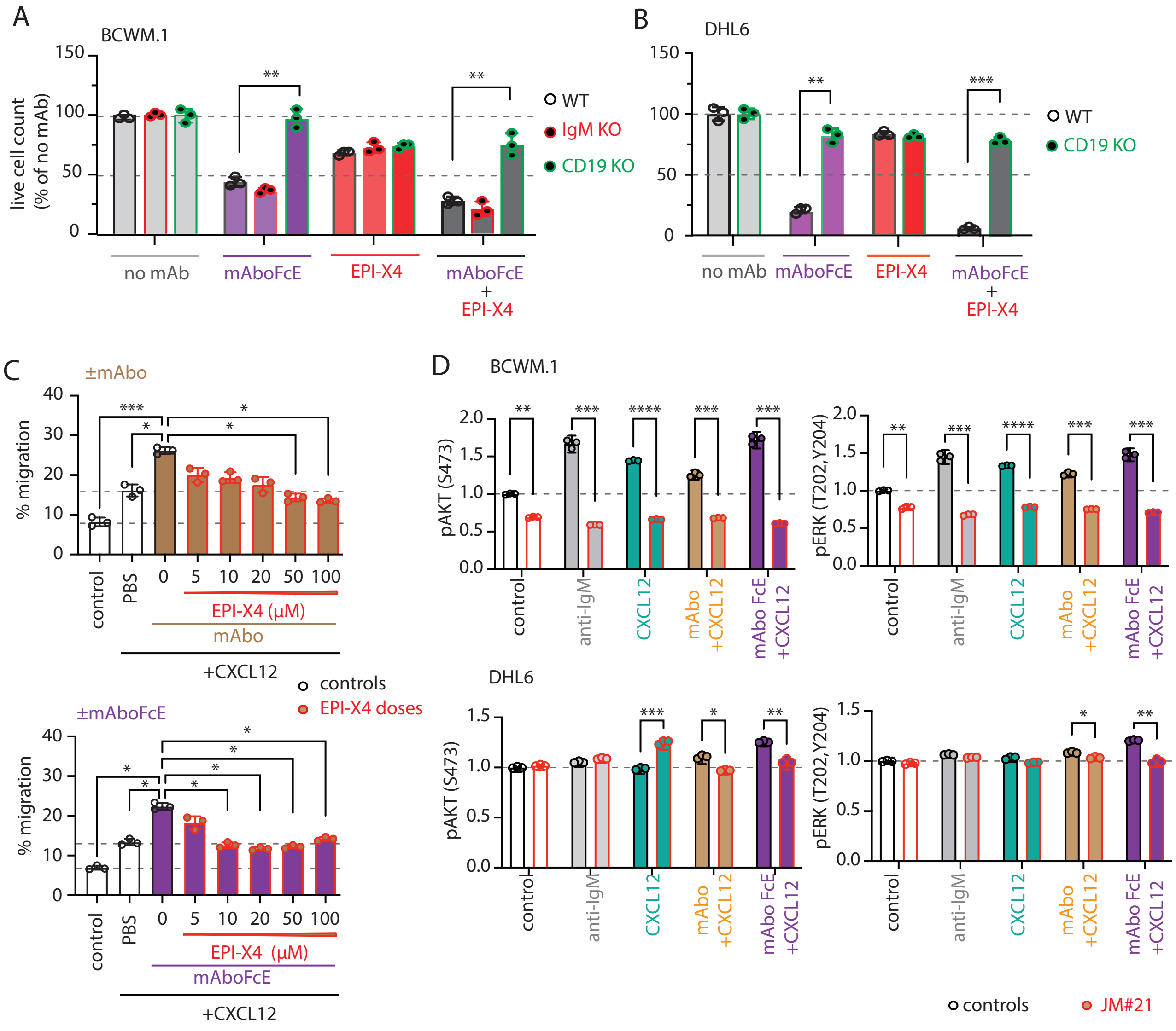

Supplement: Supplementary file 1 [file ijms-26-02024-s001.zip › ijms-3367375-Supplementary Fig.pdf]
