# Supplementary material for: CXCR4 Inhibition Enhances the Efficacy of CD19 Monoclonal Antibody-Mediated Extermination of B-Cell Lymphoma
Source: Int J Mol Sci. 2025 Feb 26;26(5):2024. doi: 10.3390/ijms26052024 (PMC11899823; doi:10.3390/ijms26052024)
Supplement: Supplementary file 1 [file ijms-26-02024-s001.zip › ijms-3367375-Supplementary Cap.pdf]

Supplementary Figure S1. Generation and analyses of CD19 KO BCWM.1 and DHL6 cells. (**A**) FACS analyses show GFP positive BCWM.1 Cas9Eco cells and loss of CD19 expression after 7, 15 and 21 days post transduction of CD19 targeting sgRNA construct. (**B**) Quantification of number of growing single cell clones of WT, CD19 KO and IgM KO BCWM.1 cells at 12, 15, 25 and 35 days of seeding in 96 well. (**C**) Representative FACS analyses of CD19 and GFP expression in a CD19 KO BCWM.1 clone compared to WT counterpart. (**D**) Sequence analyses of individual CD19 KO BCWM.1 single cell clone showing introduction of deletion and point mutation upon sgRNA targeting. Actual sequence and targeting site of the human CD19 exon2 is also depicted for comparison. (**E**) Representative FACS analyses of CD19 KO BCWM.1 clone showing unaltered expression of IgM, IgD and CXCR4 compared to WT counterpart. (**F**) Analyses of secreted IgM by ELISA of the culture supernatant of WT, CD19 KO and IgM KO BCWM.1 cells. (**G**) Representative FACS analyses of CD19 KO DHL6 cells showing loss of CD19 and unaltered IgM, IgD and CXCR4 expression compared to WT counterpart. (**H**) Same as **D**, sequence analyses of CD19 KO DHL6 clones. (**I**) Gating strategy for FACS analyses and determination of absolute cell concentration (count/μL) with reference to AccCheck counting beads.

Supplementary Figure S2. Reduced Ki-67 expression and colony growth of CD19 KO cells. (**A**) Quantification of number of cells obtained in a 4 cm^2 well from a minimum of three representative clones of CD19 and IgM KO BCWM.1 cells (left) and CD19 KO DHL6 cells (right) compared to EV (GFP) transduced WT cells. (**B**) Left, representative histogram stack of Ki-67 intracellular staining in CD19 and IgM KOs compared to WT BCWM.1 cells. Corresponding isotype controls and MFI values were shown inside the plots. Right, quantified MFI values of Ki67-staining in BCWM.1 EV, CD19 KO, and IgM KO cells with or without CD40L treatments. (**C**) Same as B, histograms and quantification of MFI of Ki67-stained in EV and CD19 KO DHL-6 cells stimulated with or without CD40L. (**D**) Representative colony image of WT (upper left), three representative CD19 KO colonies (below) and estimated colony size (right) of replated colonies from 10 days grown CFA assay. (**E**) Specific migration of BCWM.1 (left) and DHL-6 (right) cells towards varying concentrations of 15, 30, 60, 120 and 240 nM CXCL12. Data in A-E represent mean±SD of minimum three independent experiments. Data in A and D were analyzed by One-Way ANOVA followed by Dunn's multiple comparison or Mann-Whitney test. Data in B-C were analyzed by Two-Way ANOVA followed by Dunnett's multiple comparison.

Supplementary Figure S3. Development of anti-CD19 antibody mAbo and testing the functionality. (**A**) Affinity purification of recombinant anti-CD19 antibody clone mAbo secreted from transiently transduced suspension HEK239E cells. Elution profile of mAbo from HiTrap Protein G, 1mL column. (**B**) SDS-PAGE analyses of purified IgG (mAbo) in the eluted fractions compared to loading control. (**C**) Estimation of amount of purified mAbo in eluted fraction by IgG ELISA. (**D**) Left to right, FACS analyses of mAbo and mAboFcS binding to human peripheral B cells gated on CD20 and CD45R positive, and binding to BCWM.1 WT (open histograms) and CD19 KO (green filled histograms) cells. (**E**) Specific migration of DHL-6 cells at varying mAbo concentrations in presence of 60 nM CXCL12 and no CXCL12 control. (**F**) Specific migration of WT and CD19 KO BCWM.1 Cells towards 60 nM CXCL12 at varying concentration of mAbo compared to no CXCL12 control for CD19 KO cells. (**G**) Specific migration of CD19 KO BCWM.1 cells towards 60 nM CXCL12 alone and in presence of anti-CD19 clone mAbo and its Fc silent (FcS) counterpart, in comparison with other commercially available therapeutic anti-CD19 clones mAb1 and mAb2 and their FcS forms. Dashed lines in F and G represent mean of basal (with PBS) and CXCL12-induced migrations of WT cells. (**H**) Representative histogram stack of pCD79a (Y182), pERK(T202, Y204), pAKT(S473) and pPLCγ2(ϒ759) expression in DHL6 in response to anti-IgM (left panel) and CXCL12 (middle panel) treatments for 0, 5 and 10 min, followed by quantification of median fluorescence intensity (MFI) normalized to fold change compared to unstimulated controls(right panel). MFI values of each time points were indicated within the histogram plots. Data in C, E-F represent mean±SD of minimum three independent experiments. Data in E-F were analyzed by Two-Way ANOVA followed by Šídák's multiple comparison.

Supplementary Figure S4. Analyses of FACS based ADCC assay. (**A**) Representative FACS plots and gate settings of the ADCC assay with rhIL-2 stimulated NK cells. Left to right, outlines of the gating strategies for analyzing different cell populations and quantification of live cell count using AccuCheck reference beads (as depicted before in Fig S1J). FSC/SSC size gating of beads and cells, checking bead populations for their integrity and ratiometric accuracy, detection of CellTrace Red labelled NK cells and living lymphoma cells followed by identification of WT (black), IgM KO (red) and CD19 KO (green) BCWM.1 cells. Percentage of cells in each gate were indicated within the dot plots. (**B**) Mixed populations of BCWM.1 cells only. (**C**) Mixed populations of BCWM.1 cells together with NK cells. (**D**) Mixed populations of BCWM.1 cells together with NK cells in presence of mAboFcS. (**E**) Mixed populations of BCWM.1 cells together with NK cells in presence of mAbo. (**F**) Quantification of WT (black, open circles), IgM KO (red, solid circles) and CD19 KO (green, solid circles) cell count in mock ADCC assays in absence

any mAb and NK cells (as depicted in Fig S3B) and in presence of NK cells without any mAb treatment (as depicted in Fig S3C) for BCWM.1 (left panel) and DHL6 (right panel). Plots represent mean±SD of three or more replicate data normalized to no mAb control and were analyzed by Two-Way ANOVA followed by Dunnett's multiple comparison. Dashed lines represent 100 and 50% survival values.

Supplementary Figure S5. Determining the effects of EPI-X4 on mAbo induced ADCC and enhanced migration towards CXCL12. (**A**) Quantification of BCWM.1 live cell count upon treatment with different doses ranging from 1 to 100μM of CXCR4 peptide antagonist EPI-X4 and its improved derivative JM#21 compared to equivalent doses of DMSO. Cells were treated with indicated doses for 4 hours in serum free media, washed, then cultured O/N in complete media, analyzed by FACS and quantified with reference to AccuCheck beads. Data represent normalized mean±SD of minimum three replicates and analyzed by nonlinear curve fitting to estimate the IC50 value as depicted here. (**B**) Effect of JM#21 (right) and EPI-X4 (left) doses on CXCL12 induced migration of BCWM.1 cells. Data represent mean±SD of N=3 and N=6 for EPI-X4 and JM#21treatments, respectively. Dashed lines represent mean of basal (with PBS) and CXCL12-induced migrations in absence of JM#21. (**C**) Quantification of BCWM.1 WT live cell count in ADCC assay at varying concentration of mAbo (left) and JM#21(right). (**D**) Heatmaps showing the Loewe (left) and HSA (right) synergy scores of combinatorial dose-dependent effects of mAbo1 and CXCL12 on cell viability in ADCC assay. Plots represent mean of three experiments analyzed by SynergyFinder Plus tool. Synergy scores of the selected dose combination, i.e.10 μg/mL mAbo and 60 nM CXCL12 are indicated inside the plot. (**E**) Quantification of WT (black) CD19 KO (green), IgM KO (red) BCWM.1 live cell counts in ADCC assay in response to mAbo and mAboFcS in presence of CXCR4 antagonist EPI-X4 and JM#21. (**F**) Left panels, effect of JM#21 inhibition on mAboFcS and mAbo induced ADCC on BCWM.1(upper panel) and DHL6 (bottom panel). Right panels, effect of small molecule CXCR4 antagonist AMD3100 and EPI-X4 on mAboFcS and mAbo induced ADCC on BCWM.1(upper panel) and DHL6 (bottom panel). Dashed lines in C, E and F represent 100 and 50% survival values. Data in A-F represent mean±SD of minimum three independent experiments. Data in B were analyzed by One-Way ANOVA followed by Dunn's multiple comparison. Data in E and F were analyzed by Two-Way ANOVA followed by Dunnett's multiple comparison.

Supplementary Figure S6. Effects of EPI-X4 and JM#21 on mAbFcE induced ADCC, migration and phosphorylation. (**A**) Quantification of WT (black) CD19 KO (green), IgM KO (red) BCWM.1 live cell counts in ADCC assay in reponse to mAboFcE in absence and presence of 20 μM EPI-X4. (**B**) Same as B quantification of WT (black) CD19 KO (green) DHL-6 live cell counts in ADCC assay. Dashed lines in A and B represent 100 and 50% survival values. (**C**) Inhibitory effect of EPI-X4 doses on CXCL12 induced migration of BCWM.1 cells enhanced by mAbo (upper panel) and mAboFcE (lower panel) treatment. Dashed lines represent mean of basal (with PBS) and CXCL12-induced migrations in absence of mAbo or mAboFcE. (**D**) Effect of 20μM JM#21 treatment on increased pAKT (S473) and pERK (T202, Y204) in BCWM.1 (upper panels) and DHL6 (bottom panels) cells in response to stimuli- anti-IgM, mAbo + CXCL12 and mAboFcE+ CXCL12 for 5 min. Data in A-D represent mean±SD of minimum three independent experiments and were analyzed by Two-Way ANOVA followed by Dunnett's multiple comparison, except the data in C analyzed by One-Way ANOVA followed by Dunn's multiple comparison.
